# Associations of vaginal microbiota with the onset, severity, and type of symptoms of genitourinary syndrome of menopause in women

**DOI:** 10.3389/fcimb.2024.1402389

**Published:** 2024-09-24

**Authors:** Qianru Zeng, Han Shu, Heng Pan, Yonghong Zhang, Ling Fan, Yubin Huang, Li Ling

**Affiliations:** ^1^ Department of Obstetrics and Gynecology, The Second Affiliated Hospital of Chongqing Medical University, Chongqing, China; ^2^ Department of Cardiology, Renmin Hospital of Wuhan University, Wuhan, China

**Keywords:** vaginal microbiota, genitourinary syndrome of menopause (GSM), lactobacillus, microbiota dysbiosis, non-hormonal treatment

## Abstract

**Introduction:**

Genitourinary syndrome of menopause (GSM) describes the symptoms and signs resulting from the effect of estrogen deficiency on the female genitourinary tract, including genital, urinary, and sexual symptoms. However, besides estrogen deficiency, little is known about the etiology of GSM. The objective of this study was to investigate the effects of vaginal microbiota dysbiosis on the occurrence and development of GSM in perimenopausal and postmenopausal women.

**Methods:**

In total, 96 women were enrolled in this cross-sectional study and clinical data were collected. GSM symptoms were divided into three types: genital, urological, and sexual symptoms. Full-length 16S rRNA gene sequencing using the third-generation PacBio sequencing technology was performed to analyze the vaginal microbiome using vaginal swabs of non-GSM and GSM women with different types of GSM symptoms. Live Lactobacillus Capsule for Vaginal Use (LLCVU) treatment was used to verify the effects of Lactobacillus on GSM symptoms.

**Results:**

We found that 83.58% (56/67) of women experienced GSM symptoms in the perimenopausal and postmenopausal stages. Among these women with GSM, 23.21% (13/56), 23.21% (13/56), and 53.57% (30/56) had one type, two types, and three types of GSM symptoms, respectively. The richness and diversity of vaginal microbiota gradually increased from reproductive to postmenopausal women. There were significant differences in vaginal microbial community among non-GSM women and GSM women with different types of symptoms. Lactobacillus was found to be negatively associated with the onset, severity, and type of GSM while some bacteria, such as Escherichia-shigella, Anaerococcus, Finegoldia, Enterococcus, Peptoniphilus_harei, and Streptococcus, were found to be positively associated with these aspects of GSM, and these bacteria were especially associated with the types of genital and sexual symptoms in GSM women. LLCVU significantly relieved genital symptoms and improved the sexual life of GSM women in shortterm observation.

**Conclusions:**

The onset, severity, and type of GSM symptoms may be associated with changes in vaginal microbiota in perimenopausal and postmenopausal women. Vaginal microbiota dysbiosis probably contributes to the occurrence and development of GSMsymptoms, especially vaginal and sexual symptoms. Lactobacillus used in the vagina may be a possible option for non-hormonal treatment of GSM women with genital and sexual symptoms.

**Clinical Trial Registration:**

https://www.chictr.org.cn/indexEN.html, identifier ChiCTR2100044237.

## 1 Introduction

Genitourinary syndrome of menopause (GSM) is characterized by a constellation of unpleasant genital, sexual, and urinary symptoms and signs associated with estrogen deficiency that can either be self-existing or coexisting, such as vulvovaginal dryness, burning, itching, irritation, dyspareunia, reduced lubrication, decreased arousal, urgency incontinence, dysuria, or/and recurrent urinary tract infection (RUTI) (Faubion et al., 2018; Angelou et al., 2020; Cox et al., 2023). GSM is a chronic, progressive, and bothersome disorder that interferes with the quality of life of approximately half of postmenopausal and perimenopausal women worldwide (Geng et al., 2018). The North American Menopause Society statement indicates that GSM symptoms, such as vaginal dryness, urinary incontinence, and sexual dysfunction, affect 27%–84% of postmenopausal women, and that over half of women with vaginal symptoms reported that these symptoms adversely affected their sexual health and quality of life (2020). Thus, GSM requires early recognition and appropriate treatment to preserve the urogenital health of postmenopausal and perimenopausal women worldwide (Cox et al., 2023). However, besides the association with the decrease of serum estrogen levels in women, little is known about the etiology of GSM (Mitchell et al., 2021).

The onset and progression of GSM are complicated. To date, some studies have focused on vaginal microbiota in healthy and GSM individuals (Geng et al., 2020; Shardell et al., 2021). Studies showing an association between the symptoms of GSM (vulvovaginal atrophy or dryness) and the decrease in Lactobacillus bacteria in the vaginas of postmenopausal women suggested that vaginal colonization with lactobacilli might mediate the development of GSM (Hummelen et al., 2011; Brotman et al., 2014). However, some studies reported that there was no significant association between GSM symptoms and vaginal dominance by the Lactobacillus species in postmenopausal women (Mitchell et al., 2017, Mitchell et al., 2018). Thus, the potential relationship between the onset and development of GSM and the dysbiosis of vaginal microbiota is still full of ambiguity.

Studies associated with vaginal microbiota and health usually use partial variable regions of the 16S rRNA gene for sequencing, which makes it impossible to obtain accurate information at the species level (Zhang et al., 2020). The selection of v3-v4 regions for the 16S rRNA gene sequence might bias the results (Chen et al., 2018; Diao et al., 2021). Some bacteria of the same genus are associated with both health and disease, suggesting different pathogenic potentials of species in the same genus (Chen et al., 2018; Diao et al., 2021). Therefore, it is of great significance to deeply sequence microorganisms at the species level (Diao et al., 2021). Based on the third-generation PacBio sequencing technology, studies have demonstrated that full-length bacterial 16S rRNA gene sequencing can provide accurate information at the species level and detailed identification of complex microbial communities with high throughput (Singer et al., 2016; Wagner et al., 2016; Johnson et al., 2019).

Thus, in this cross-sectional study, we examined the vaginal microbiota in reproductive, perimenopausal, and postmenopausal women using full-length bacterial 16S rRNA gene sequencing. We aimed to reveal the vaginal microbiota of women of different ages as well as their differences in perimenopausal and postmenopausal women with or without GSM symptoms to determine the occurrence and development of vaginal microbiota dysbiosis in persons with GSM. These results may offer a foundation for exploring the etiology of and effective treatment strategies for GSM.

## 2 Materials and methods

### 2.1 Ethics approval and informed consent

This study was ethically approved by the Ethics Committee of the Second Affiliated Hospital of Chongqing Medical University (issuing number: 2020-226) and registered in the Chinese Clinical Trial Registry (No. ChiCTR2100044237). The study was in compliance with the Helsinki Declaration. Written informed consent was obtained from all women before enrollment.

### 2.2 Study participants

Women were recruited from patients attending the clinic for physical examination in the Second Affiliated Hospital of Chongqing Medical University in China, from March 2022 to September 2022. A total of 96 women ranging in age from 18 to 79 years old participated in the study. Participants were enrolled and classified after examining their medical history and finishing gynecological examinations. Participants were required to complete one-on-one questionnaires conducted by trained investigators. Gynecological examinations were performed by a specialized gynecologist. The contents of the questionnaire included age, body mass index (BMI), education, profession, smoking status, health status, gravidity, parity, menstruation, menopausal status, history of drug use, operations, and diseases. The covariate data from the interviews and examinations including age and smoker status (Shardell et al., 2021), which may influence vaginal microbiota, were analyzed and corrected. Confounders including sexual activity (within 48 hours), vaginitis, the use of vaginal drugs/agents (within 3 months), exogenous hormone use (within 3 months), and diseases that may affect vaginal health status were excluded.

Common symptoms of GSM were divided into three types: i) genital symptoms including itching, burning, irritation, dryness, pain, and leukorrhea; ii) urological symptoms including urgent, frequent, and painful urination, incontinence, dysuria, and RUTI; iii) sexual symptoms including dyspareunia, reduced lubrication, post-coital bleeding, decreased arousal, orgasm, desire, and loss of libido and arousal. Objectively, the severity of GSM was evaluated by the vaginal symptoms score (VSS) (Davila et al., 2003), vaginal health index score (VHIS) (Davila et al., 2003), female sexual function index (FSFI) (Rosen et al., 2000), international consultation on incontinence questionnaire - short form (ICIQ-SF) (Tamanini et al., 2004), and menopause quality of life questionnaire (MENQOL). The VSS and the FSFI, ICIQ-SF, and MENQOL scores were obtained from questionnaires, while the VHIS was evaluated by an experienced gynecologist through gynecological examination.

The inclusion criteria of this study were as follows: i) woman older than 16 years old; ii) had a history of sexual life; iii) systemic health. The exclusion criteria were as follows: i) received gynecological treatment in the last 3 months; ii) used antibiotics, antifungal, or immunosuppressant medication within the last 3 months; iii) were lactating or pregnant; iv) diagnosed with systemic disease that may affect vaginal health status; v) having gynecological inflammation; vi) had irregular menstrual bleeding.

### 2.3 VSS and VHIS forms used for the assessment of vulvovaginal symptoms and signs in women

The VSS and VHIS forms were used to evaluate the vulvovaginal symptoms and signs, respectively (**Supplementary Table 1**) (Davila et al., 2003; Areas et al., 2019). The presence and severity of each recorded symptom (VSS) and sign (VHIS) of GSM were assigned a score from 0 (none/not present) to 3 (severe) (Davila et al., 2003). The total VSS and VHIS were calculated by the sum of individual VSS and VHIS divided by 5, respectively. A VHIS or VSS indicates a worse vaginal physical condition.

### 2.4 FSFI form used for the assessment of female sexual function in women

The FSFI is a multidimensional self-report instrument for the assessment of female sexual function (Rosen et al., 2000). The FSFI form is a 19-item questionnaire, and individual items are assigned to six separate domains of female sexual function: desire, arousal, lubrication, orgasm, satisfaction, and pain (**Supplementary Table 2A**). The range of each item score is from 0/1 to 5. Individual domain scores were obtained by adding the scores of the individual items that comprise the domain and multiplying the sum by each domain factor (**Supplementary Table 2B**). The total scores were obtained by adding the six domain scores. A higher score of FSFI indicates better sexual function.

### 2.5 ICIQ-SF form used for the assessment of urinary incontinence in women

The ICIQ-SF is a simple, brief, and condition-specific quality-of-life questionnaire for patients with urinary incontinence (**Supplementary Table 3**). It contains four questions which evaluate the frequency, severity, and influence of urinary incontinence by scoring, plus a set of eight self-diagnosis items related to the situations or causes of urinary incontinence experienced by the patients (Avery et al., 2004; Tamanini et al., 2004). The total scores of the ICIQ-SF were calculated as the sum of the individual scores of questions 1, 2, and 3.

### 2.6 MENQOL form used for the assessment of the quality of life in women

The MENQOL form was used for the assessment of the global quality of life of postmenopausal and perimenopausal women (**Supplementary Table 4**). The MENQOL form is a 30-item questionnaire, and individual items are assigned to four domains: vasomotor, psychosocial, physical, and sexual life. The presence and severity of each item were assigned a score from 0 (not at all bothered) to 6 (extremely bothered) which were evaluated by patient’s self-perception. The total and each domain scores were calculated by the sum of individual items.

### 2.7 Study design

**Figure 1:**
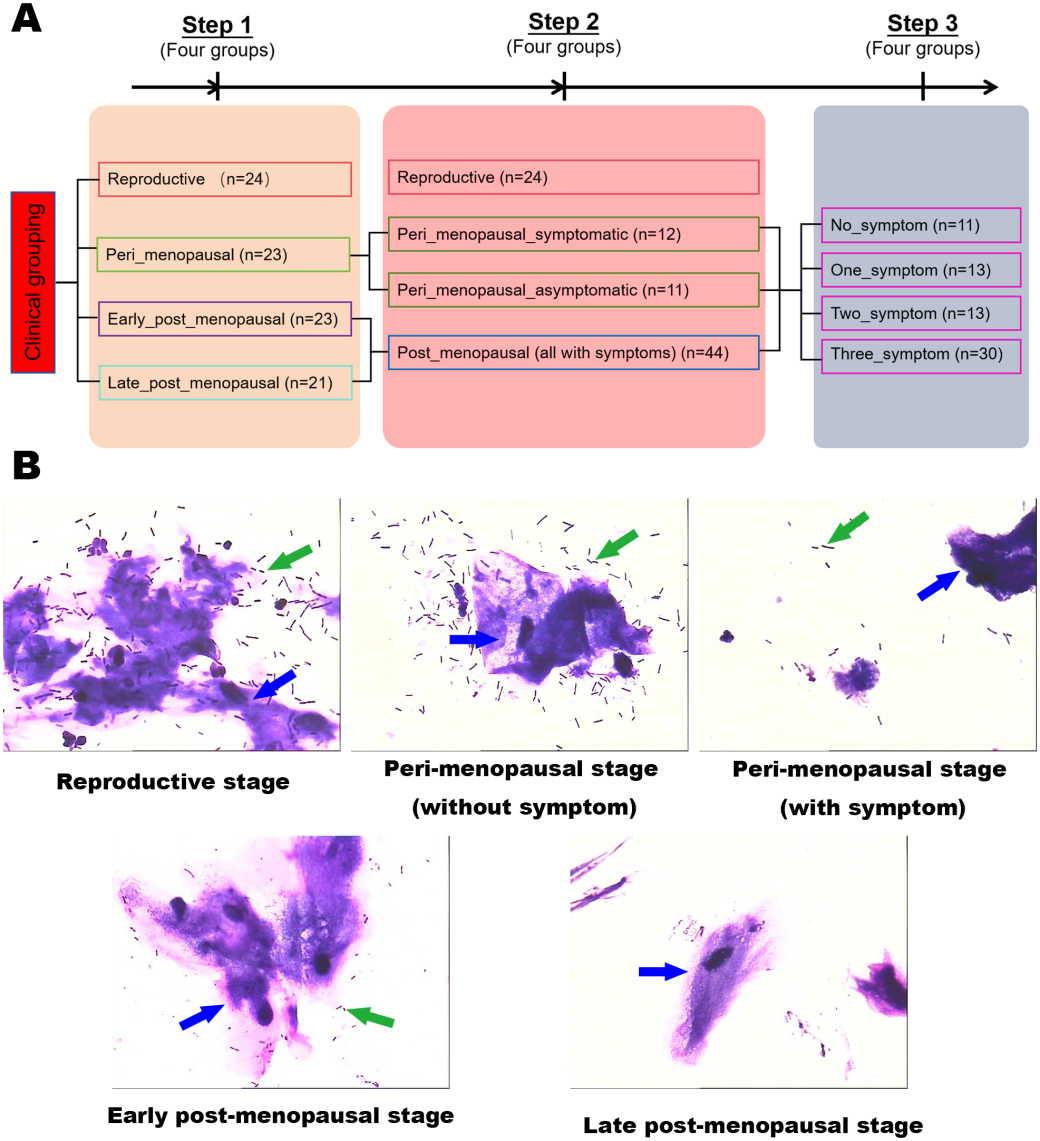
Experimental grouping and schematic. **(A)** The grouping and analytical flowchart of the study. **(B)** Gram stains of vaginal secretion samples in the reproductive, peri_menopausal, early_post_menopausal, and late_post_menopausal groups (×1000). The green arrows indicate Lactobacillus and the blue arrows indicate cornified epithelium.

The grouping and analytical flowchart of the study are shown in **Figure 1A**.

To reveal the vaginal microbiota in women of different ages, the participants were first divided into four groups according to the ages of the women and the duration of menopause, namely, the reproductive (n=24), peri_menopausal (n=23), early_post_menopausal (n=23), and late_post_menopausal (n=21) groups. The reproductive group was defined as women in the child-bearing stage and younger than 40 years old. The peri_menopausal group was defined as women older than 40 years old and in the perimenopause stage with elevated levels of follicle-stimulating hormone (FSH, between 10 mIU/mL ~ 40 mIU/mL) and an irregular menstrual cycle. The early_post_menopausal group was defined as women who had been experiencing menopause for ≤10 years. The late_post_menopausal group was defined as women who had been experiencing menopause for >10 years.

Then, to explore the difference in vaginal microbiota between GSM and non-GSM women in perimenopausal and postmenopausal women, the participants were also divided into four groups according to the perimenopausal and postmenopausal women with or without GSM symptoms, namely, the reproductive (n=24), peri_menopausal_asymptomatic (n=11), peri_menopausal_symptomatic (n=12), and post_menopausal groups (n=44). The definition of the reproductive group was the same as aforementioned. The peri_menopausal_asymptomatic group was defined as women without GSM symptoms in the perimenopausal phase. The peri_menopausal_symptomatic group was defined as women with GSM symptoms in the perimenopausal phase. The post_menopausal group was defined as women with GSM symptoms in the postmenopausal phase as we found that all the postmenopausal women in our study had GSM symptoms.

GSM mainly encompasses three types of manifestations, namely, genital, urological, and sexual symptoms. To further determine whether vaginal microbiota are associated with the types of GSM symptoms, the participants in the perimenopausal and postmenopausal phases were finally further divided into four groups, namely, the no_symptom (n=11), one_symptom (n=13), two_symptom (n=13), and three_symptom (n=30) groups. The no_symptom group was defined as women in the perimenopausal and postmenopausal phases without GSM symptoms. The one_symptom group was defined as women in the perimenopausal and postmenopausal phases with one type of GSM symptom. The two_symptom group was defined as women in the perimenopausal and postmenopausal phases with two types of GSM symptoms. The three_symptom group was defined as women in the perimenopausal and postmenopausal phases with three types of GSM symptoms.

### 2.8 Collection of vaginal secretion samples

Individuals were sampled before the onset of treatment in “ChiCTR2100044237”. Vaginal secretion samples were obtained from women without sexual activity, vaginal douching, vaginal examination, or the use of vaginal medications and lubrication at least 48 hours prior to the gynecological examination. Vaginal secretion samples were obtained from the posterior vaginal fornix of the women using four sterile swabs. Two swabs were immediately used for the smear, and the slides were stained by Gram and observed under an optical microscope (Olympus Corporation, Tokyo, Japan) by a trained laboratory technician. The other two swabs were put into a sterile cryopreservation tube and stored in a liquid nitrogen container until they were processed for full-length 16S rRNA gene sequencing.

### 2.9 DNA extraction and PCR amplification

Microbial community genomic DNA from the vaginal secretion samples was extracted and qualified. The full-length bacterial 16S rRNA genes were amplified by PCR using the universal primers 27F (5’-AGRGTTYGATYMTGGCTCAG-3’) and 1492R (5’-RGYTACCTTGTTACGACTT-3’). Primers were tailed with PacBio barcode sequences. The PCR amplification was performed as follows: 1 cycle of denaturation for 3 min at 95°C followed by 28 cycles of denaturation for 30 s at 95°C, annealing for 30 s at 60°C, extension for 45 s at 72°C, a single extension for 10 min at 72°C, and then end at 10°C (ABI GeneAmp^®^ 9700 PCR thermocycler, CA, USA). PCR reactions were performed in triplicate. PCR products were purified using AMPure^®^ PB beads (Pacific Biosciences, CA, USA) and quantified with a Quantus™ Fluorometer (Promega, WI, USA).

### 2.10 DNA library construction and sequencing using the PacBio Sequel II System

DNA library was constructed using the SMRTbell^®^ Express Template Prep Kit 2.0 (Pacifc Biosciences, CA, USA) according to PacBio’s instruction and sequenced on the PacBio Sequel II System (Pacifc Biosciences, CA, USA) by Majorbio Bio-Pharm Technology Co. Ltd. (Shanghai, China).

### 2.11 Data processing of vaginal microbiota

Raw reads sequenced by the PacBio Sequel II System were processed using SMRTLink analysis software (version 8.0) to obtain demultiplexed circular consensus sequence (CCS) reads with a minimum of three full passes and 99% sequence accuracy. The CCS reads were barcode-identified and length-filtered, and sequences with lengths between 1000 bp and 1800 bp were kept.

The optimized-CCS reads were de-noised using divisive amplicon denoising algorithm 2 (DADA2) and the CCS plugin in the Qiime2 (version 2020.2) pipeline with recommended parameters. The sequences denoised by DADA2 are usually called amplicon sequence variants (ASVs).

To minimize the influence of sequencing depth on alpha and beta diversity assays, the number of sequences of each sample was rarefied to 11,869, which was the smallest number of sequences of all samples. With the cutoff of 11,869, the average Good’s coverage of all samples was 99.98%. Based on the SILVA 16S rRNA database (v138), taxonomic assignments of the ASVs were performed using Naive Bayes in Qiime2.

### 2.12 Treatment protocol of the Live Lactobacillus Capsule for Vaginal Use for GSM women

In total, 29 women with GSM undergoing Live Lactobacillus Capsule for Vaginal Use (LLCVU) treatment between April 2021 and July 2022 at the Second Affiliated Hospital of Chongqing Medical University in China were retrospectively analyzed. This retrospective analysis was ethically approved by the Ethics Committee of the Second Affiliated Hospital of Chongqing Medical University (issuing number: 2020-226). The patients received vaginal administration of the LLCVU (containing 0.25×10^6^ CFU of Lactobacillus; INNER MONGOLIA Shuangqi Pharmaceutical Co., Ltd., Hohhot, Inner Mongolia, China) before sleeping once a day for 10 consecutive days as the course of treatment. According to the LLCVU use instructions, the LLCVU was placed deeply into women’s vaginas using a finger cot after they cleaned their external genitalia each day. Vaginal douching and the use of antibiotics and other agents or drugs such as vaginal lubricant, vaginal moisturizer, and hormone treatment, were not allowed in the process of the treatment. The patients were followed up at 2 weeks after the first day of treatment, and the symptoms and signs of GSM were evaluated.

### 2.13 Data analysis of vaginal microbiota

Bioinformatic analysis of the vaginal microbiota was performed using the Majorbio Cloud platform (https://cloud.majorbio.com). R package MaAsLin 2 3.3.1 was used to correct covariables. Based on the ASV information, rarefaction curves and alpha diversity index were calculated using Mothur v1.30.1. Principal coordinate analysis (PCoA) was conducted using R package ggpubr 3.3.1, and the beta diversity in different groups was further analyzed and compared using the vegan package and PERMANOVA test. R package MaAsLin 2 3.3.1 was used to identify the significantly abundant taxa of bacteria in different groups. Canonical correspondence analysis (CCA) and Spearman’s correlation analysis were performed to investigate the relationship between vaginal bacterial community structure and GSM symptoms. Random forest algorithm (RF) and receiver operating characteristic (ROC) analyses were conducted using the randomForest and plotROC packages (version 3.3.1), respectively, for the prediction model analysis. To establish the model for GSM prediction, RF analysis consisting of 500 trees was first used to determine the important vaginal bacterial species that caused differences between the GSM and non-GSM groups, and then these important species were selected as biomarkers for distinguishing samples in different groups and used to construct a prediction model. Additionally, approximately 10% to 20% of the samples were excluded from each group before RF analysis to verify the accuracy of the prediction model later. After that, ROC curves were further used to assess the accuracy of the prediction model built by the biomarkers for GSM diagnosis. The metagenomic function was predicted by a Phylogenetic Investigation of Communities by Reconstruction of Unobserved States (PICRUSt2) using R package vegan 3.3.1 (Douglas et al., 2020) based on the ASV representative sequences.

### 2.14 Statistical analysis

Statistical analysis was conducted using SPSS 22.0 software (IBM, NY, USA). Data are presented as mean ± standard deviation (SD) or medians and ranges. The nonparametric Kruskal-Wallis test and Mann-Whitney U test were used for multiple-group and two-group comparisons, respectively. Fisher’s exact test was used for the comparison of categorical variables. Statistical significance was set at P<0.05.

## 3 Results

### 3.1 Clinical characteristics of study subjects

A total of 96 women participated in the study. However, 91 of them were available for analysis as the other 5 women had gynecological inflammation. The main demographic characteristics of the women of different ages in the reproductive, peri_menopausal, early_ post_menopausal and late_post_menopausal groups are described in **Table 1**. There were significant differences in BMI, education, profession, gravidity, and parity among these groups (P <0.05).

**Table 1:** Clinical characteristics of women of different ages (n=91).

Characteristics	Reproductive (n=24)	Peri_menopausal (n=23)	Early_post_ menopausal (n=23)	Late_post_ menopausal (n=21)	P value
**Age(Median, range)**	29.8 (18~39)	46.7 (40~52)	55.0 (49~61)	67.7 (55~79)	0.000^a^
**BMI(Median, range)**	20.6 (17.7~25.8)	22.1 (18.6~30.8)	22.5 (18.9~27.5)	21.8 (17.7~26.7)	0.037
Education(N, %)					0.000^a^
Primary school	0 (0)	1 (4.3)	6 (26.1)	9 (42.9)	
Secondary school	2 (8.3)	9 (39.1)	12 (52.2)	8 (38.1)	
College graduate	21 (87.5)	13 (56.5)	5 (21.7)	4 (19.0)	
Postgraduate	1 (4.2)	0 (0)	0 (0)	0 (0)	
Profession(N, %)					0.000^a^
Unemployed	0 (0)	2 (8.7)	5 (21.7)	12 (57.1)	
Worker	0 (0)	3 (13.0)	6 (26.1)	6 (28.6)	
Farmer	0 (0)	2 (8.7)	2 (8.7)	1 (4.8)	
Office clerk	22 (91.7)	15 (65.2)	7 (30.4)	2 (9.5)	
Professionals	1 (4.2)	1 (4.3)	1 (4.3)	0 (0)	
Freelance work	1 (4.2)	0 (0)	2 (8.7)	0 (0)	
**Gravidity (Median, range)**	1.2 (0~5)	2.8 (1~6)	2.6 (1~7)	2.8 (1~6)	0.000^a^
**Parity (Median, range)**	0.6 (0~2)	1.4 (1~2)	1.2 (1~3)	1.3 (1~3)	0.000^a^
GSM symptoms (N, %)					
Genital symptoms	3 (12.5)	6 (26.1)	20 (87.0)	21 (100.0)	0.000^b^
Urological symptoms	6 (25.0)	4 (17.4)	16 (69.6)	13 (61.9)	0.000^b^
Sexual symptoms	0 (0)	8 (34.8)	20 (87.0)	21 (100.0)	0.000^b^
**MENOQL (mean ± SD)**	–	7.124 ± 2.353	10.104 ± 3.563	11.736 ± 2.273	0.000^a^
Vasomotor	–	2.014 ± 1.345	2.377 ± 1.742	1.333 ± 0.775	0.058^a^
Psychosocial	–	1.342 ± 0.531	1.562 ± 0.462	1.863 ± 0.799	0.032^a^
Physical	–	1.449 ± 0.399	1.916 ± 0.576	2.079 ± 0.591	0.000^a^
Sexual	–	2.319 ± 1.312	4.246 ± 1.936	6.460 ± 1.496	0.000^a^
**FSFI (mean ± SD)**	30.933 ± 2.074	25.700 ± 7.163	14.987 ± 7.1034	6.214 ± 5.686	0.000^a^
Desire	4.850 ± 0.611	4.226 ± 0.798	2.530 ± 0.745	1.457 ± 0.486	0.000^a^
Arousal	5.138 ± 0.681	3.887 ± 1.431	2.374 ± 1.304	0.657 ± 1.002	0.000^a^
Lubrication	5.863 ± 0.177	4.683 ± 1.615	2.674 ± 1.538	0.786 ± 1.292	0.000^a^
Orgasm	4.550 ± 0.438	4.035 ± 1.337	2.330 ± 1.425	0.686 ± 1.074	0.000^a^
Satisfaction	4.883 ± 0.425	4.313 ± 0.772	2.887 ± 1.065	1.867 ± 0.924	0.000^a^
Pain	5.650 ± 0.476	4.557 ± 1.657	2.191 ± 1.511	0.762 ± 1.283	0.000^a^
**VSS (mean ± SD)**	0.025 ± 0.068	0.087 ± 0.146	0.461 ± 0.415	1.010 ± 0.496	0.000^a^
Dryness	0	0.043 ± 0.209	0.696 ± 0.703	1.238 ± 0.768	0.000^a^
Soreness	0.042 ± 0.204	0	0.130 ± 0.344	0.429 ± 0.507	0.000^a^
Irritation	0	0	0.478 ± 0.593	1.238 ± 0.700	0.000^a^
Dyspareunia	0	0.174 ± 0.388	0.913 ± 0.848	2.000 ± 1.000	0.000^a^
Vaginal discharge	0.083 ± 0.282	0.217 ± 0.422	0.087 ± 0.288	0.143 ± 0.359	0.500^a^
**VHIS (mean ± SD)**	0.050 ± 0.122	0.313 ± 0.335	1.217 ± 0.443	1.638 ± 0.459	0.000^a^
Vaginal secretions	0.125 ± 0.338	0.609 ± 0.656	1.783 ± 0.422	2.0 ± 0.447	0.000^a^
Vaginal epithelial integrity	0	0.043 ± 0.209	0.696 ± 0.635	1.429 ± 0.598	0.000^a^
Vaginal epithelial surface thickness	0	0.348 ± 0.573	1.304 ± 0.635	1.810 ± 0.750	0.000^a^
Vaginal color	0.042 ± 0.204	0.348 ± 0.487	1.261 ± 0.541	1.810 ± 0.512	0.000^a^
Vaginal PH	0.083 ± 0.282	0.217 ± 0.422	1.043 ± 0.825	1.143 ± 0.910	0.000^a^
**ICIQ-SF (mean ± SD)**	1.417 ± 2.552	0.609 ± 2.105	2.391 ± 3.929	3.000 ± 4.690	0.168^a^
ICIQ-SF- question 1	0.333 ± 0.637	0.130 ± 0.458	0.652 ± 1.112	0.667 ± 1.111	0.175^a^
ICIQ-SF- question 2	0.500 ± 0.885	0.174 ± 0.576	0.609 ± 0.941	0.667 ± 0.966	0.220^a^
ICIQ-SF- question 3	0.583 ± 1.060	0.304 ± 1.105	1.130 ± 2.001	1.667 ± 2.763	0.174^a^

a Kruskal-Wallis nonparametric test.

b Fisher’s exact test.

VSS, vaginal symptoms score; VHIS, vaginal health index score; FSFI, female sexual function index; ICIQ-SF, international consultation on incontinence questionnaire - short form; MENQOL, menopause quality of life questionnaire. SD, standard deviation.

In the peri_menopausal group, 52.17% (12/23) of the women had GSM symptoms, including genital, urological and/or sexual symptoms, while 100% (44/44) of postmenopausal women in the early_ and late_post_menopausal groups had GSM symptoms. There was a significant difference in the incidence of GSM among the four groups (**Table 1**, P <0.05).

### 3.2 Clinical characteristics of women with GSM

In our study, it was found that 83.58% (56/67) of the women experienced GSM symptoms in the perimenopausal and postmenopausal stages. The characteristics of GSM symptoms in each group are described in **Table 1**. Among these women with GSM, 53.57% (30/56) of them had three types of GSM symptoms, while 23.21% (13/56) of them had one type, and 23.21% (13/56) of them had two types of GSM symptoms, in the perimenopausal and postmenopausal stages. Then, we divided the women with GSM symptoms into the one_symptom, two_symptom and three_symptom groups for further analysis. We found that the women in the two_symptom group only had genital and sexual symptoms (100%), while the women in the one_symptom group had either genital (30.77%), urological (23.08%), or sexual (46.15%) symptoms.

To evaluate the severity of GSM symptoms in the peri_menopausal, early_ post_menopausal, and late_post_menopausal groups, VSS, VHIS, FSFI, ICIQ-SF, and MENQOL questionnaires were used. We found that the total VSSs, VHISs, and MENOQL scores were significantly higher, while the total FSFI scores were significantly lower, in the early_ and late_post_menopausal groups than in the peri_menopausal group (P<0.05). There were no significant differences in the total VHISs, and FSFI, ICIQ-SF, and MENQOL scores between the early_post_menopausal and late_post_menopausal groups (P>0.05). However, compared to the early_post_menopausal group, the total VSS was significantly higher in the late_post_menopausal group (**Table 1**, P<0.05). Interestingly, there was no significant difference in the total scores of ICIQ-SF in the reproductive, peri_menopausal, early_ post_menopausal, and late_post_menopausal groups (**Table 1**, P>0.05).

The results of the vaginal secretion smear showed that the inhibition of microbial communities in the vagina gradually increased from the perimenopausal to the postmenopausal stage (**Figure 1B**). There was an obvious difference in the number of vaginal Lactobacillus between women with GSM symptoms and women without symptoms in the perimenopausal stage. The number of vaginal Lactobacillus in the women with GSM symptoms in the perimenopausal stage was similar to that in the women with GSM symptoms in the postmenopausal stage, while the number of vaginal Lactobacillus in the women without GSM symptoms in the perimenopausal stage was similar to that in the reproductive women. These results showed that vaginal microbiota might be associated with GSM symptoms in women, and the vaginal microbiota in women of different ages and in women with or without GSM was subsequently explored by full-length 16S rRNA gene sequencing.

### 3.3 Vaginal microbiota in women of different ages

**Figure 2:**
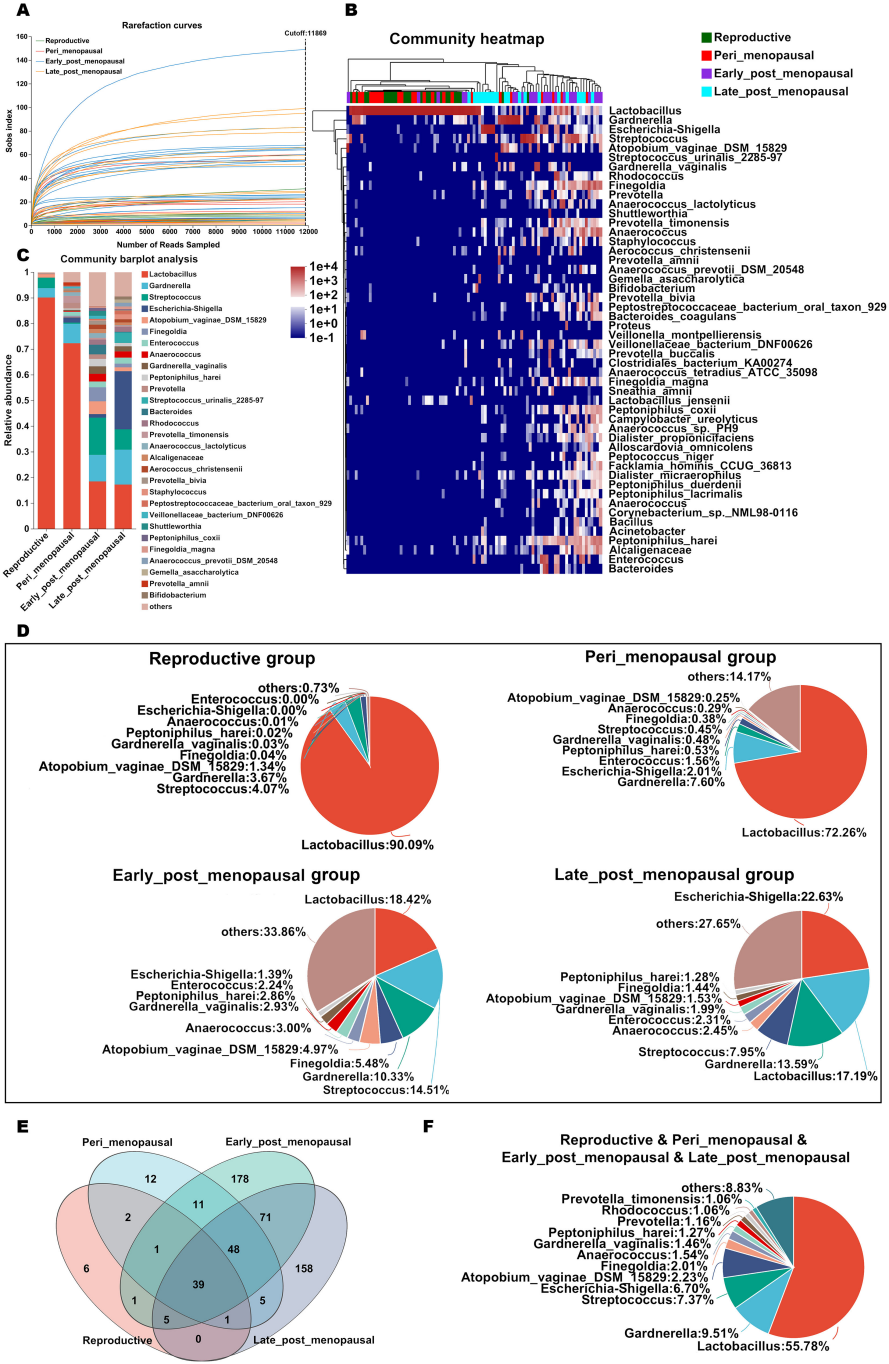
The relative abundance of the microbial community in the vagina in women of different ages. **(A)** The species richness of the vaginal microbiota of each sample was estimated by rarefaction curve analysis based on Sob indexes. **(B, C)** Relative abundance of microbial taxa in the vagina of the reproductive, peri_menopausal, early_post_menopausal, and late_post_menopausal groups at the species level (top 50) is shown by heatmap analysis **(B)** and compared using barplot analysis **(C)**. Each sample in the heatmap is shown as a vertical bar and the different colors of each cell represent different relative abundances. The high abundance and low abundance are highlighted in red and blue, respectively. The horizontal bars on the top indicate the reproductive (green), peri_menopausal (red), early_post_menopausal (purple), and late_post_menopausal (blue) groups, and dendrograms reflect hierarchical clustering. **(D)** The relative abundance of the top 10 classified species in the vagina of the four groups is shown using a pieplot. **(E)** The common and different microbial species in the vagina of the four groups are shown using a Venn diagram. **(F)** The common microbial species in the vagina of the four groups are shown using a pieplot (abundance proportions of bacteria >0.01).

A total of 3,979,926 sequences with an average length of 1,513 bp per sample were generated after quality filtering and 6,611 ASVs were acquired. The high-quality sequences belonging to 1 kingdom were classified into 50 phyla, 136 classes, 277 orders, 434 families, 858 genera, and 1,645 species through bioinformatic processing. The species richness of the vaginal microbiota of each sample was estimated using rarefaction curve analysis (**Figure 2A**). The results showed that enough sequencing data were generated and 11,869 sequences per sample were adequate for capturing the microbial diversity in all samples as the Good’s coverage of all samples reached above 99.8% and the rarefaction curves were relatively plateaued.

To investigate details about the microbial community at the species level in the vaginas of women of different ages, the relative abundance of the top 50 classified species in all samples was demonstrated as a hierarchically clustered heatmap (**Figure 2B**), and the average relative abundance of species in the reproductive, peri_menopausal, early_post_menopausal, and late_post_menopausal groups was different (**Figure 2C**). It was found that Lactobacillus was the dominant bacterial species in the reproductive and peri_menopausal groups, but not in the early_post_menopausal and late_post_menopausal groups (**Figures 2B, C**). The relative abundance of the top 10 classified species of the four groups is shown in **Figure 2D**. The common and different species in the four groups were further analyzed using a Venn diagram (**Figure 2E**) and pieplot (**Figure 2F**). The Venn diagram showed 39 of the common species in the four groups (**Figure 2E**) and those of which the abundance proportions were greater than 0.01 are shown in Figure F. There were 6, 12, 178, and 158 species in the reproductive, peri_menopausal, early_ post_menopausal and late_post_menopausal groups, respectively (**Figure 2E**). These results showed that Lactobacillus was the most represented bacteria in the reproductive, peri_menopausal, and early_post_menopausal groups (**Figures 2C, D**), while Escherichia-shigella was the most represented bacteria in the late_post_menopausal group (**Figures 2C, D**). Compared to reproductive women, the Lactobacillus proportion in the vagina gradually decreased from the perimenopausal women to late postmenopausal women, while the proportion of other bacteria, such as Escherichia-shigella and Gardnerella, gradually increased (**Figures 2C, D**).

**Figure 3:**
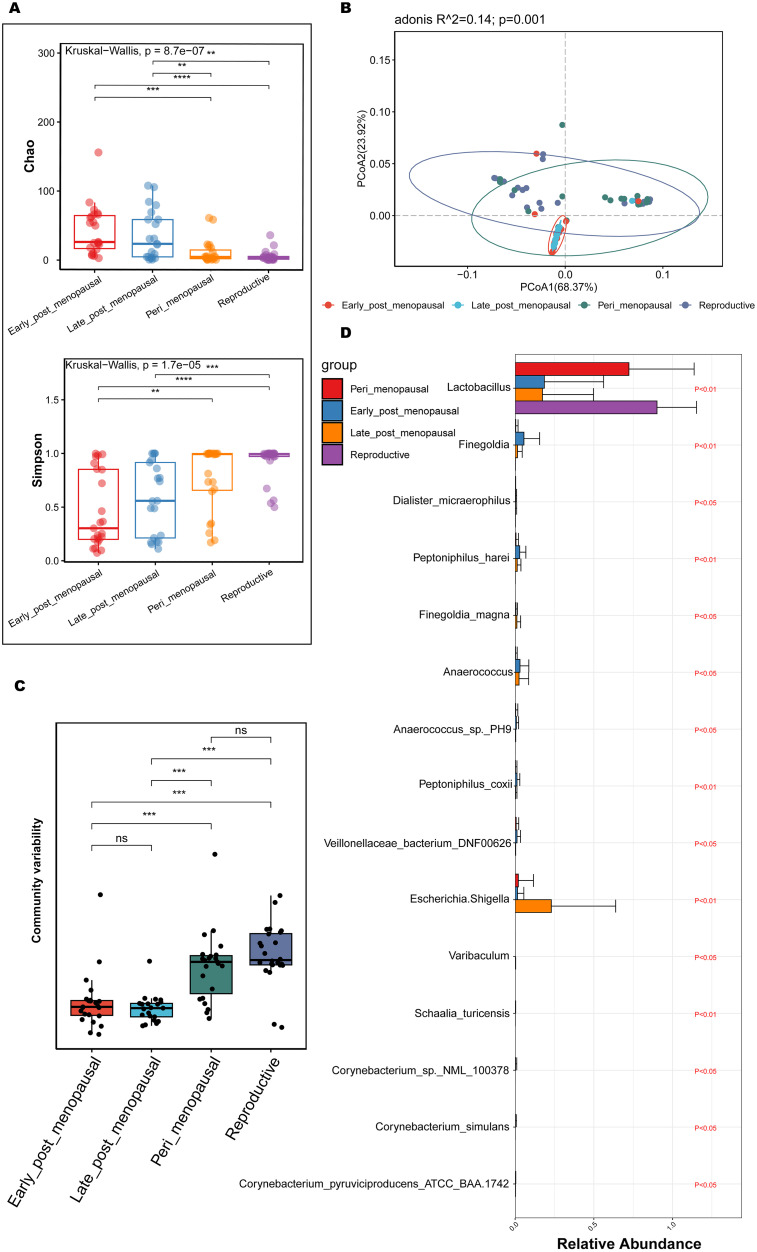
Comparisons of alpha and beta diversity in the reproductive, peri_menopausal, early_ post_menopausal, and late_post_menopausal groups. **(A)** Microbial richness is represented by Chao indices and microbial diversity is represented by Simpson indices. **(B, C)** PCoA based on Bray-Curtis distance showing the variation **(B)** and differences **(C)** of microbial community structure in the vagina in the reproductive, peri_menopausal, early_ post_menopausal, and late_post_menopausal groups. **(D)** Discriminatory bacteria contributing to significant intergroup differences among the four groups presented using MaAsLin 2. **P < 0.01, ***P < 0.001, ****P < 0.0001. Each dot with a different color represents a sample in the corresponding group, and the 95% confidence intervals of the samples’ distribution of each group are visualized by ellipses.

To compare the richness and diversity of the microbial community in the vaginas of women of different ages, the alpha diversity (Chao and Simpson index) and beta diversity (PCoA) were analyzed in the reproductive, peri_menopausal, early_ post_menopausal, and late_post_menopausal groups. Compared to the reproductive group, the Chao indexes were significantly higher (**Figure 3A**, P<0.01), while the Simpson indexes were significantly lower, in the early_ post_menopausal and late_post_menopausal groups (**Figure 3A**, P<0.01). Compared to the peri_menopausal group, the Chao indexes were significantly higher in the early_post_menopausal and late_post_menopausal groups (**Figure 3A**, P<0.01), while the Simpson index was significantly lower in the early_ post_menopausal group (**Figure 3A**, P<0.01). Beta-diversity analysis also showed that there were significant differences among the four groups (**Figures 3B, C**, P<0.01). The community variability in the reproductive and peri_menopausal groups was significantly higher than that in the early_ and late_post_menopausal groups (**Figure 3C** and **Supplementary Table 6-1**, P<0.01). However, there were no significant differences in the alpha and beta diversities between the reproductive and peri_menopausal groups (**Figures 3A, C**, P>0.05). There were also no significant differences in the alpha and beta diversities between the early_ and late_post_menopausal groups (**Figures 3A, C**, P>0.05).To further explore which bacteria made the greatest contributions to the observed intergroup difference, MaAsLin 2 was performed in the above four groups. There were 15 species presenting significant intergroup differences in the reproductive, peri_menopausal, early_ post_menopausal and late_post_menopausal groups (**Figure 3D**, all P<0.05). Anaerococcus_sp._PH9, Corynebacterium_sp._NML_100378, Corynebacterium_simulans, Dialister_micraerophilus, Peptoniphilus_harei, Schaalia_turicensis, Anaerococcus, Finegoldia, Peptoniphilus_coxii, Corynebacterium_pyruviciproducens_ATCC_BAA.1742, and Veillonellaceae_bacterium_DNF00626 had an increased abundance in the early_post_menopausal group (**Figure 3D**). In contrast, only Lactobacillus had an increased abundance in the reproductive group (**Figure 3D**), while Escherichia-Shigella, Finegoldia_magna, and Varibaculum had an increased abundance in the late_post_menopausal group (**Figure 3D**).

These results demonstrate that the richness and diversity of vaginal microbiota were significantly higher in the postmenopausal women than in the reproductive and perimenopausal women. The changes in vaginal microbiota may be a gradual process from the perimenopausal stage to the postmenopausal stage.

### 3.4 Differences of vaginal microbiota between non-GSM and GSM women

**Figure 4:**
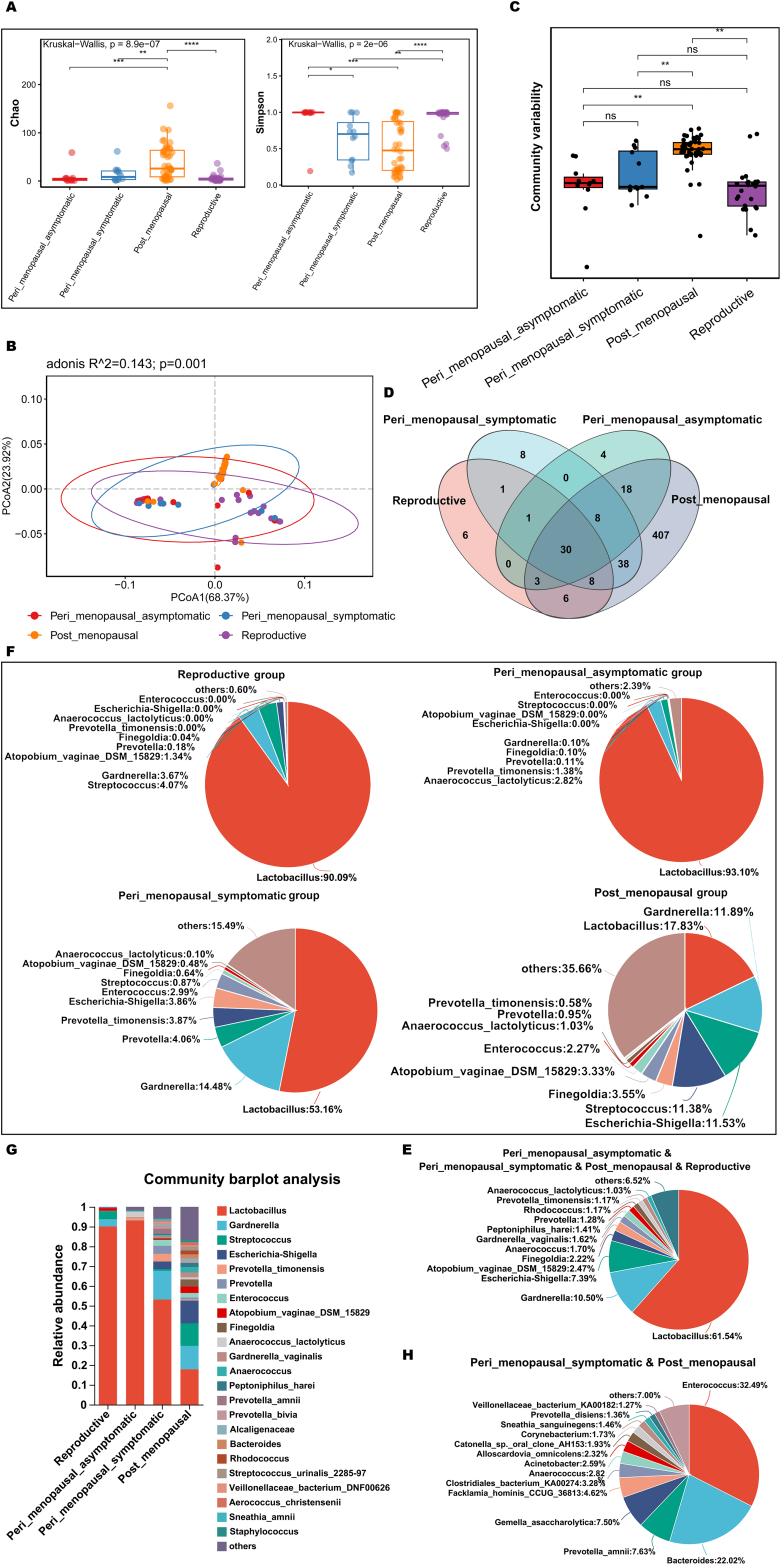
Comparisons of vaginal microbial communities in non-GSM and GSM women. **(A)** Microbial richness is represented by Chao indices and microbial diversity is represented by Simpson indices in the reproductive, peri_menopausal_asymptomatic, peri_menopausal_symptomatic and post_menopausal groups are shown. **(B, C)** The variation **(B)** and differences **(C)** of microbial community structure in the vagina in the above four groups are presented using PCoA based on Bray-Curtis distance. **(D)** The common and different microbial species in the vagina of the four groups are shown using a Venn diagram. **(E)** The common microbial species in the vagina of the four groups are shown using a pieplot (abundance proportions of bacteria>0.01). **(F)** The relative abundance of the top 10 classified species in the vagina of the four groups is shown using a pieplot. **(G)** The relative abundance of microbial taxa in the vagina of the four groups at the species level is compared. **(H)** The common microbial species only in the vagina of the peri_menopausal_symptomatic and post_menopausal groups are shown using a pieplot (abundance proportions of bacteria>0.01). *P < 0.05, **P < 0.01, ***P < 0.001, ****P < 0.0001.

To explore and compare the richness and diversity of microbial community in vagina of non-GSM and GSM women in the perimenopausal and postmenopausal stages, alpha diversity (Chao and Simpson index) and beta diversity (PCoA) were analyzed in the reproductive, peri_menopausal_asymptomatic, peri_menopausal_symptomatic and post_menopausal groups. It was found that the Chao indexes of non-GSM women were significantly lower in the reproductive and peri_menopausal_asymptomatic groups than those of the GSM women in the post_menopausal group (**Figure 4A**, P<0.01), while the Simpson indexes of non-GSM women were significantly higher in the reproductive and peri_menopausal_asymptomatic groups than those of the GSM women in the peri_menopausal_symptomatic and post_menopausal groups (**Figure 4A**, P<0.05). However, there were no significant differences in the Chao and Simpson indexes between the reproductive and peri_menopausal_asymptomatic groups (**Figure 4A**, P>0.05). There was also no significant difference in the Simpson index between the peri_menopausal_symptomatic and post_menopausal groups (**Figure 4A**, P>0.05). Beta-diversity analysis also showed that there were significant differences among the four groups (**Figures 4B, C**, P<0.01). The community variability of GSM women in the post_menopausal group was significantly higher than those of non-GSM women in the reproductive and peri_menopausal_asymptomatic groups (**Figure 4C** and **Supplementary Table 6-2**, P<0.01). These results demonstrate that the richness and diversity of vaginal microbiota are higher in GSM women than in non-GSM women, and the occurrence of GSM might be associated with the changes in the vaginal microbial community in the perimenopausal and postmenopausal women.

To know more details about the differences in vaginal microbiota between non-GSM and GSM women in the perimenopausal and postmenopausal stages, the type and relative abundance of species in the vagina in the above four groups were determined. There were 30 common species in the four groups (**Figure 4D**), of which those with abundance proportions greater than 0.01 are shown in **Figure 4E**. There were 6, 4, 8, and 407 species in the reproductive, peri_menopausal_asymptomatic, peri_menopausal_symptomatic, and post_menopausal groups, respectively (**Figure 4D**). The relative abundance of vaginal bacteria in the four groups was shown (top 10 species, **Figure 4F**) and compared (abundance proportions>0.01, **Figure 4G**). The vaginal bacteria of the women in the peri_menopausal_symptomatic group were similar to those of the women in the post_menopausal group to an extent while the vaginal bacteria of the women in the peri_menopausal_asymptomatic group were similar to those of the women in the reproductive group. There were 38 species of vaginal bacteria only existing in the peri_menopausal_symptomatic and post_menopausal groups (abundance proportions>0.01, **Figure 4H**).

**Figure 5:**
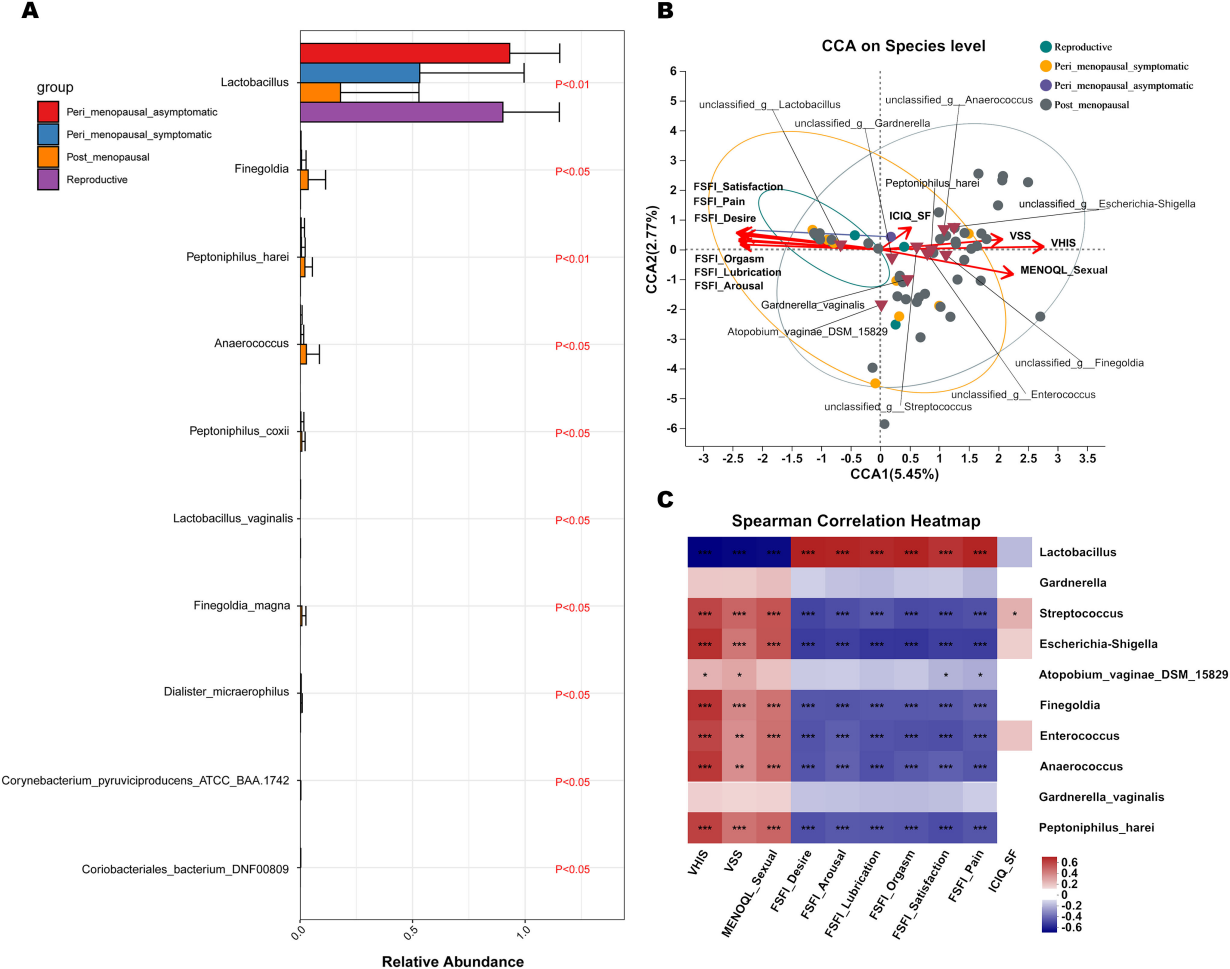
Differential species analysis and correlation analysis between vaginal microbiota and clinical characteristics in non-GSM and GSM women. **(A)** MaAsLin 2 was performed to identify discriminatory bacteria among the reproductive, peri_menopausal_asymptomatic, peri_menopausal_symptomatic and post_menopausal groups. **(B, C)** CCA **(B)** and heatmap of Spearman’s correlation analysis **(C)** between the vaginal microbiota and clinical characteristics in the four groups reveal the potentially multivariate correlation of the clinical characteristics of GSM and primary microbial community. In the CCA, each dot with different color represents a sample in the corresponding group, and the 95% confidence intervals of samples’ distribution of each group are visualized by ellipses. A greater distance between samples indicates a stronger dissimilarity in microbial structure. Brown inverted triangles represent the top 10 microbial species. Clinical characteristic variables (VSS, VHIS, ICIQ_SF, MENOQL_sexual, FSFI_desire, FSFI_arousal, FSFI_lubrication, FSFI_orgasm, FSFI_satisfaction, and FSFI_pain) are indicated by vectors in red, and the vectors of VSS/VHIS, ICIQ-SF, and MENOQL_sexual/FSFI are used for the assessment of the genital, urological and sexual symptoms, respectively The length of the vector is used for the interpretation of this correlation, and a longer vector indicates a greater correlation with the bacterium and sample distribution, while the angle between the vectors reflects the association of the clinical variables, and an acute angle indicates a positive association and an obtuse angle indicates a negative association. In the heatmap of Spearman’s correlation analysis, the correlation coefficient r is between −1 and 1; r < 0 represents negative correlation, r > 0 represents positive correlation. *P < 0.05, **P < 0.01, ***P < 0.001. (CCA, canonical correlation analysis; VSS, vaginal symptoms score; VHIS, vaginal health index score; FSFI, female sexual function index; MENQOL, menopause quality of life questionnaire; ICIQ-SF, international consultation on incontinence questionnaire - short form).

MaAsLin 2 was further performed to identify discriminatory bacteria that made a greater contribution to the observed differences in the reproductive, peri_menopausal_asymptomatic, peri_menopausal_symptomatic, and post_menopausal groups. There were 10 species presenting significant intergroup differences in the four groups (**Figure 5A**, all P<0.05). Dialister_micraerophilus, Peptoniphilus_harei, Anaerococcus, Peptoniphilus_coxii, Finegoldia, Finegoldia_magna, and Corynebacterium_pyruviciproducens_ATCC_BAA.1742 had an increased abundance in women with GSM in the post_menopausal group (**Figure 5A**), while only Coriobacteriales_bacterium_DNF00809 had an increased abundance in women with GSM in the peri_menopausal_symptomatic group (**Figure 5A**). In contrast, Lactobacillus had an increased abundance in women without GSM in the reproductive and peri_menopausal_asymptomatic groups (**Figure 5A**). These results demonstrate that the bacteria mentioned above might be important biomarkers for the identification of GSM and non-GSM women in the perimenopausal and postmenopausal stages.

CCA and a heatmap of Spearman’s correlation analysis were performed to reveal the potential multivariate correlations of the clinical characteristics of GSM and primary microbial community (**Figure 5B, C**). In the CCA, the length of the vector is used for the interpretation of this correlation, and a longer vector indicates a greater correlation with bacterium and sample distribution, while the angle between vectors reflects the association of clinical variables. An acute angle indicates a positive association and an obtuse angle indicates a negative association. Additionally, a greater distance between samples indicates a stronger dissimilarity in microbial structure. The vectors of VSS/VHIS, ICIQ-SF, and MENOQL_sexual/FSFI were used for the assessment of genital, urological, and sexual symptoms, respectively. The results showed that the distribution of samples in the post_menopausal group was generally in accordance with the VSS, VHIS, and MENOQL_sexual vectors but in the opposite direction of all the FSFI vectors (**Figure 5B**), whereas the distribution of samples in the reproductive and peri_menopausal_asymptomatic groups was generally in accordance with all the FSFI vectors but in the opposite direction of the VSS, VHIS and MENOQL_sexual vectors (**Figure 5B**). Furthermore, 4 of 12 samples in the peri_menopausal_symptomatic group were in accordance with the samples in the post_menopausal group, while 7 of 12 samples in the peri_menopausal_symptomatic group were in accordance with the samples in the reproductive and peri_menopausal_asymptomatic groups (**Figure 5B**). The distribution of these samples was generally in accordance with the comparison of the onset and severity of GSM symptoms among these groups. CCA and Spearman’s correlation analysis also showed a correlation between GSM symptoms and the relative abundance of the top 10 species in the vagina. The vectors of VSS (r^2^ = 0.4002, P<0.01), VHIS (r^2^ = 0.6994, P<0.01), MENOQL_sexual (r^2^ = 0.4815, P<0.01), FSFI_Desire (r^2^ = 0.5141, P<0.01), FSFI_Arousal (r^2^ = 0.4838, P<0.01), FSFI_Lubrication (r^2^ = 0.5136, P<0.01), FSFI_Orgasm (r^2^ = 0.5027, P<0.01), FSFI_Satisfaction (r^2^ = 0.5403, P<0.01), and FSFI_Pain (r^2^ = 0.5154, P<0.01) were significantly correlated with Lactobacillus, Escherichia-shigella, Anaerococcus, Finegoldia, Enterococcus, Peptoniphilus_harei, and Streptococcus in the vagina (**Figures 5B, C**; P<0.01), while these vectors had no significant correlation with Gardnerella and Gardnerella_vaginalis (**Figures 5B, C**; P>0.05). It was found that Lactobacillus was significantly correlated with all the FSFI vectors positively and with the VSS, VHIS, and MENOQL_sexual vectors negatively (**Figures 5B, C**; P<0.01), while Escherichia-shigella, Anaerococcus, Finegoldia, Enterococcus, Peptoniphilus_harei, and Streptococcus were significantly correlated with the VSS, VHIS, and MENOQL_sexual vectors positively and with all the FSFI vectors negatively (**Figures 5B, C**; P<0.01). Moreover, the correlations mentioned above remained after correcting for covariates. However, all bacteria distributions except Streptococcus showed no significant correlation with the ICIQ_SF vector (r^2^ = 0.0546, P>0.05; **Figures 5B, C**). These results demonstrate that Lactobacillus may be negatively associated with the occurrence and severity of GSM symptoms while the other bacteria mentioned above may be positively associated with these aspects of GSM symptoms, especially the genital and sexual symptoms.

### 3.5 Vaginal microbiota in GSM women with different types of symptoms

To further determine whether vaginal microbiota are associated with the types of GSM symptoms, the women in the perimenopausal and postmenopausal phases were divided into the no_symptom, one_symptom, two_symptom, and three_symptom groups, and the main clinical characteristics of women in those four groups are shown in **Supplementary Table 5**. There were significant differences in the scores of MENOQL, FSFI, VSS, VHIS, and ICIQ-SF among those four groups (**Supplementary Table 5**, all P <0.05).

**Figure 6:**
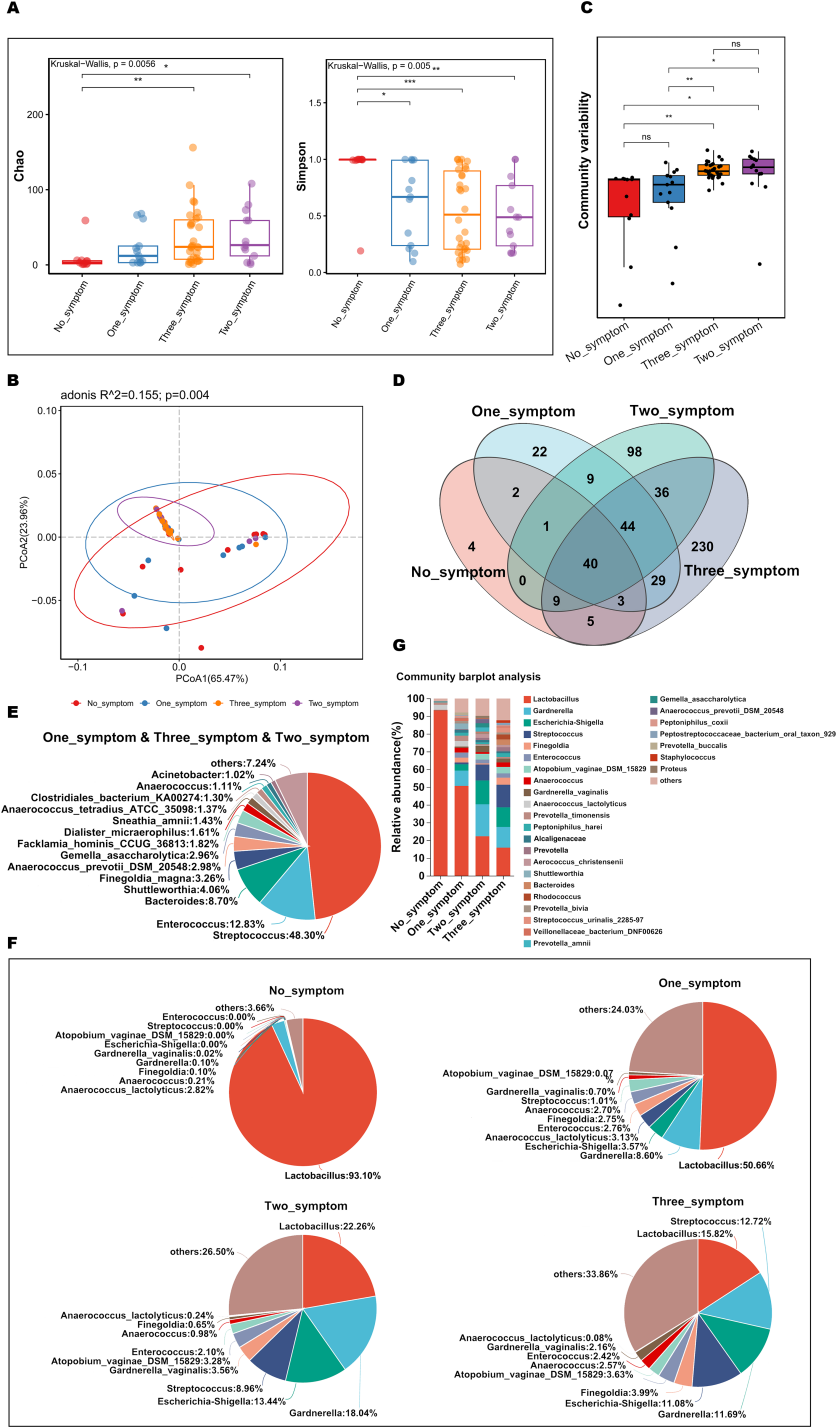
Comparisons of the vaginal microbial community in GSM women with different types of symptoms. **(A)** Microbial richness represented by Chao indices and microbial diversity represented by Simpson indices in the no_symptom, one_symptom, two_symptom, and three_symptom groups are shown. **(B, C)** The variation **(B)** and differences **(C)** of microbial community structure in the vagina in the above four groups are presented using PCoA based on Bray-Curtis distance. **(D)** The common and different microbial species in the vagina of the four groups are shown using a Venn diagram. **(E)** The common microbial species in the vagina of the one_symptom, two_symptom and three_symptom groups are shown using a pieplot (abundance proportions of bacteria>0.01). **(F)** The relative abundance of the top 10 classified species in the vagina of the four groups is shown using a pieplot. **(G)** The relative abundance of microbial taxa in the vagina of the four groups at the species level is compared. *P < 0.05, **P < 0.01, ***P < 0.001. Each dot with different color represents a sample in the corresponding group, and the 95% confidence intervals of samples’ distribution of each group are visualized by ellipses.

To explore and compare the richness and diversity of the microbial community in the vaginas of GSM women with different types of GSM symptoms, alpha diversity (Chao and Simpson indexes) and beta diversity (PCoA) were analyzed in the no_symptom, one_symptom, two_symptom and three_symptom groups. Compared to the no_symptom group, the Chao indexes were significantly higher in the two_symptom and three_symptom groups (**Figure 6A**, P<0.05), while the Simpson indexes were significantly lower in the one_symptom, two_symptom, and three_symptom groups (**Figure 6A**, P<0.05). However, the differences in the Chao index between the no_symptom and one_symptom groups were not significant (**Figure 6A**, P>0.05). Beta-diversity analysis showed that there were significant differences among the four groups (**Figures 6B, C**, P<0.05). The community variability in the two_symptom and three_symptom groups were significantly higher than that in the no_symptom and one_symptom groups (**Figure 6C** and **Supplementary Table 6-3**, P<0.05). However, there were no significant differences between the two_symptom and three_symptom groups, or no_symptom and one_symptom groups (**Figure 6C**, P>0.05).

To investigate details about the differences in vaginal microbiota in GSM women with different types of symptoms, the variety and relative abundance of species in the vagina in the above four groups were determined. There were 44 common species in the GSM women in the one_symptom, two_symptom, and three_symptom groups (**Figure 6D**), of which those with abundance proportions greater than 0.01 are shown in **Figure 6E**. There were 21, 98, and 229 species in the one_symptom, two_symptom, and three_symptom groups, respectively (**Figure 6D**). The relative abundance of vagina bacteria in the four groups was shown (top 10 species, **Figure 6F**) and compared (abundance proportions>0.01, **Figure 6G**). Compared to the one_symptom group, the vaginal microbiota of the women in the two_symptom group was much closer to that of the women in the three_symptom group (**Figures 6F, G**). The types of GSM symptoms gradually increased as the proportion of Lactobacillus gradually decreased and other bacteria, such as Streptococcus and s:Atopobium_vaginae_DSM_15829, increased in the vagina (**Figures 6F, G**).

**Figure 7:**
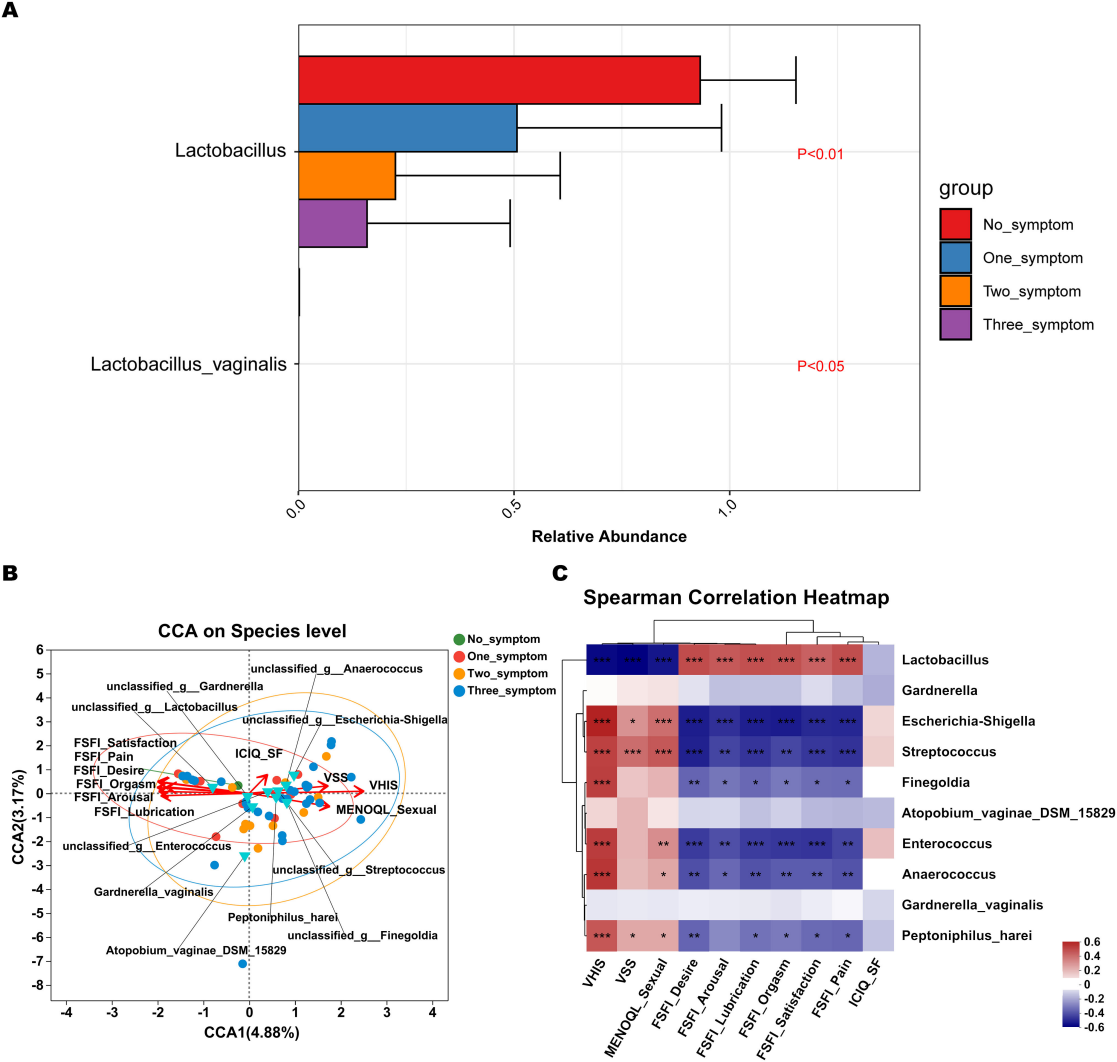
Differential species analysis and correlation analysis between vaginal microbiota and clinical characteristics in GSM women with different types of symptoms. **(A)** MaAsLin 2 was performed to identify discriminatory bacteria in the no_symptom, one_symptom, two_symptom, and three_symptom groups. **(B)** CCA between the vaginal microbiota and clinical characteristics in the four groups. In the CCA, each dot with a different color represents a sample in the corresponding group, and the 95% confidence intervals of samples’ distribution of each group are visualized by ellipses. A greater distance between samples indicates a stronger dissimilarity in microbial structure. Blue inverted triangles represent the top 10 microbial species. Clinical characteristic variables (VSS, VHIS, ICIQ_SF, MENOQL_sexual, FSFI_desire, FSFI_arousal, FSFI_lubrication, FSFI_orgasm, FSFI_satisfaction, and FSFI_pain) are indicated by vectors in red. The length of the vector is used for the interpretation of this correlation, and a longer vector indicates a greater correlation with bacterium and sample distribution, while the angle between vectors reflects the association of clinical variables, and an acute angle indicates a positive association and an obtuse angle indicates a negative association. **(C)** Heatmap of Spearman’s correlation analysis between the vaginal microbiota and clinical characteristics in the four groups is shown. The correlation coefficient r is between −1 and 1; r < 0 represents negative correlation, r > 0 represents positive correlation. *P < 0.05, **P < 0.01, ***P < 0.001 (CCA, canonical correlation analysis; VSS, vaginal symptoms score; VHIS, vaginal health index score; FSFI, female sexual function index; MENQOL, menopause quality of life questionnaire; ICIQ-SF, international consultation on incontinence questionnaire - short form).

Discriminatory bacterium analysis showed there were two bacterial species presenting significant intergroup differences in the no_symptom, one_symptom, two_symptom, and three_symptom groups (**Figure 7A** P<0.05). Lactobacillus and Lactobacillus_vaginalis had an increased abundance in non-GSM women in the no_symptom group (**Figure 7A**, P<0.05). Compared to the no_symptom group, the relative abundance of Lactobacillus and Lactobacillus_vaginalis was significantly higher in the one_symptom, two_symptom, and three_symptom groups (all P<0.05). These results demonstrate that Lactobacilli might play important roles in the onset, severity, and type of GSM symptoms and may be pivotal biomarkers for the identification between GSM women and non-GSM women.

CCA showed that the samples of perimenopausal and postmenopausal women with two types of GSM symptoms (genital and sexual symptoms) in the two_symptom group and those with three types of GSM symptoms in the three_symptom group were generally in accordance with the VSS, VHIS, and MENOQL_sexual vectors but in the opposite direction of all the FSFI vectors (**Figure 7B**). Moreover, the correlation between the samples and the VSS, VHIS, and MENOQL_sexual vectors was generally closer in the three_symptom group than in the two_symptom group. Interestingly, we found that the distribution of the samples of GSM women with only urological symptoms in the one_symptom group was close to that of the non-GSM women in the no_symptom group, while the distribution of the samples of GSM women with only genital symptoms in the one_symptom group was close to that of GSM women in the two_symptom and three_symptom groups (**Figure 7B**). These results demonstrated that the distribution of the samples was in accordance with the clinical manifestation of women with GSM. The vectors of VSS (r^2^ = 0.3108, P<0.01), VHIS (r^2^ = 0.6501, P<0.01), MENOQL_sexual (r^2^ = 0.3287, P<0.01), FSFI_Desire (r^2^ = 0.4123, P<0.01), FSFI_Arousal (r^2^ = 0.3321, P<0.01), FSFI_Lubrication (r^2^ = 0.395, P<0.01), FSFI_Orgasm (r^2^ = 0.3795, P<0.01), FSFI_Satisfaction (r^2^ = 0.4184, P<0.01), and FSFI_Pain (r^2^ = 0.4057, P<0.01), which represent genital and sexual symptoms, were found to be significantly correlated with vaginal Lactobacillus, Escherichia-shigella, Streptococcus, etc. (**Figures 7B, C**; P<0.05). It was found that Lactobacillus correlated with all the FSFI vectors positively and with the VSS, VHIS, and MENOQL_sexual vectors negatively, while other bacteria such as Escherichia-shigella Streptococcus, etc., correlated with VSS, VHIS and MENOQL_sexual vectors positively and with FSFI vectors negatively (**Figures 7B, C**; P<0.01). Moreover, the correlations mentioned above remained after correcting for covariates. However, the ICIQ_SF vector, which represents urological symptoms, showed no significant correlation with these bacteria (r^2^ = 0.0569, P>0.05; **Figures 7B, C**).

These results further demonstrate that Lactobacillus may be negatively associated with the onset and severity of GSM symptoms, while Escherichia-shigella, Streptococcus, etc., may be positively associated with these aspects of GSM symptoms, and these bacteria may be mainly associated with the types of genital and sexual symptoms in GSM women.

### 3.6 Marker species analysis between non-GSM and GSM women with different types of symptoms

**Figure 8:**
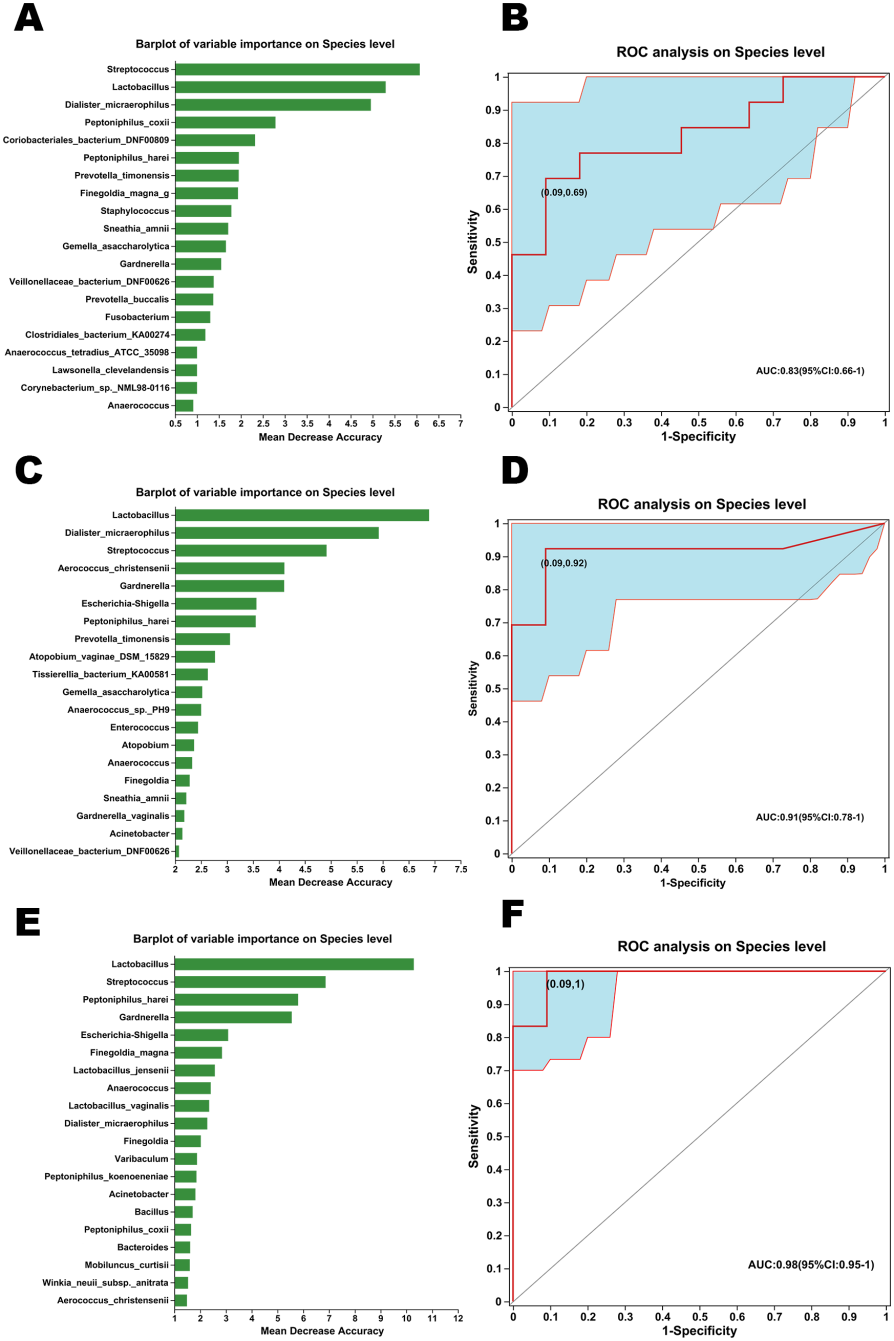
Marker species analysis between non-GSM and GSM women with different types of symptoms. **(A)** The variable importance of bacteria in the vagina on the species level was analyzed by the random forest algorithm for the differentiation between the no_symptom and one_symptom groups. **(B)** Receiver operating characteristic (ROC) analysis of distinguishing between the no_symptom group and the one_symptom group using detected species was conducted using the area under the curve (AUC). **(C)** The variable importance of bacteria in the vagina on the species level was analyzed by the random forest algorithm for the differentiation between the no_symptom and two_symptom groups. **(D)** ROC analysis of distinguishing between the no_symptom group and two_symptom group using detected species was conducted using the AUC. **(E)** The variable importance of bacteria in the vagina on the species level was analyzed by the random forest algorithm for the differentiation between no_symptom and three_symptom groups. **(F)** ROC analysis of distinguishing between the no_symptom group and three_symptom group using detected species was conducted using the AUC.

Random forest algorithm and ROC analysis were conducted for the exploration of species markers between non-GSM and GSM women with different types of symptoms in the no_symptom, one_symptom, two_symptom, and three_symptom groups. The top 20 important bacterial species for the discrimination between the no_symptom and one_symptom groups are shown in **Figure 8A**. A combination of three species, Streptococcus, Lactobacillus, and Dialister_micraerophilus, showed optimal important features for the differentiation of these two groups as determined by the random forest algorithm and were selected as specific microbial biomarkers and used for the building of a prediction model. The sensitivity and specificity of the model were verified by the ROC assay. The model achieved an optimal area under the curve (AUC) value of 0.83 (sensitivity: 69%, specificity: 0.91%; **Figure 8B**). The top 20 important bacteria for the discrimination between the no_symptom and two_symptom groups are shown in **Figure 8C**. A combination of two species, Lactobacillus and Dialister_micraerophilus, showed optimal important features for the differentiation of these two groups as determined by the random forest algorithm and were selected as specific microbial biomarkers and used for the building of a prediction model. The model achieved an AUC value of 0.91 (sensitivity: 92%, specificity: 0.91%; **Figure 8D**). Finally, the top 20 important bacteria for the discrimination between no_symptom and three_symptom groups are shown in **Figure 8E**. A combination of 15 species showed optimal important features for the differentiation of these two groups as determined by the random forest algorithm, and the top 5 were Lactobacillus, Streptococcus, Peptoniphilus_harei, Gardnerella, and Escherichia-shigella, which were also selected as specific microbial biomarkers and used for the building of a prediction model. The model achieved an AUC value of 0.98 (sensitivity: 100%, specificity: 0.91%; **Figure 8F**). These results demonstrate that the diagnosis models built using specific vaginal bacteria between non-GSM women and GSM women are comparatively accurate, especially between non-GSM women and GSM women with more than one type of symptoms, which might be used for the prediction of the onset, severity, and type of GSM.

### 3.7 The changes in the potential functional capacity of the vaginal microbiome between non-GSM and GSM patients with different types of symptoms

**Figure 9:**
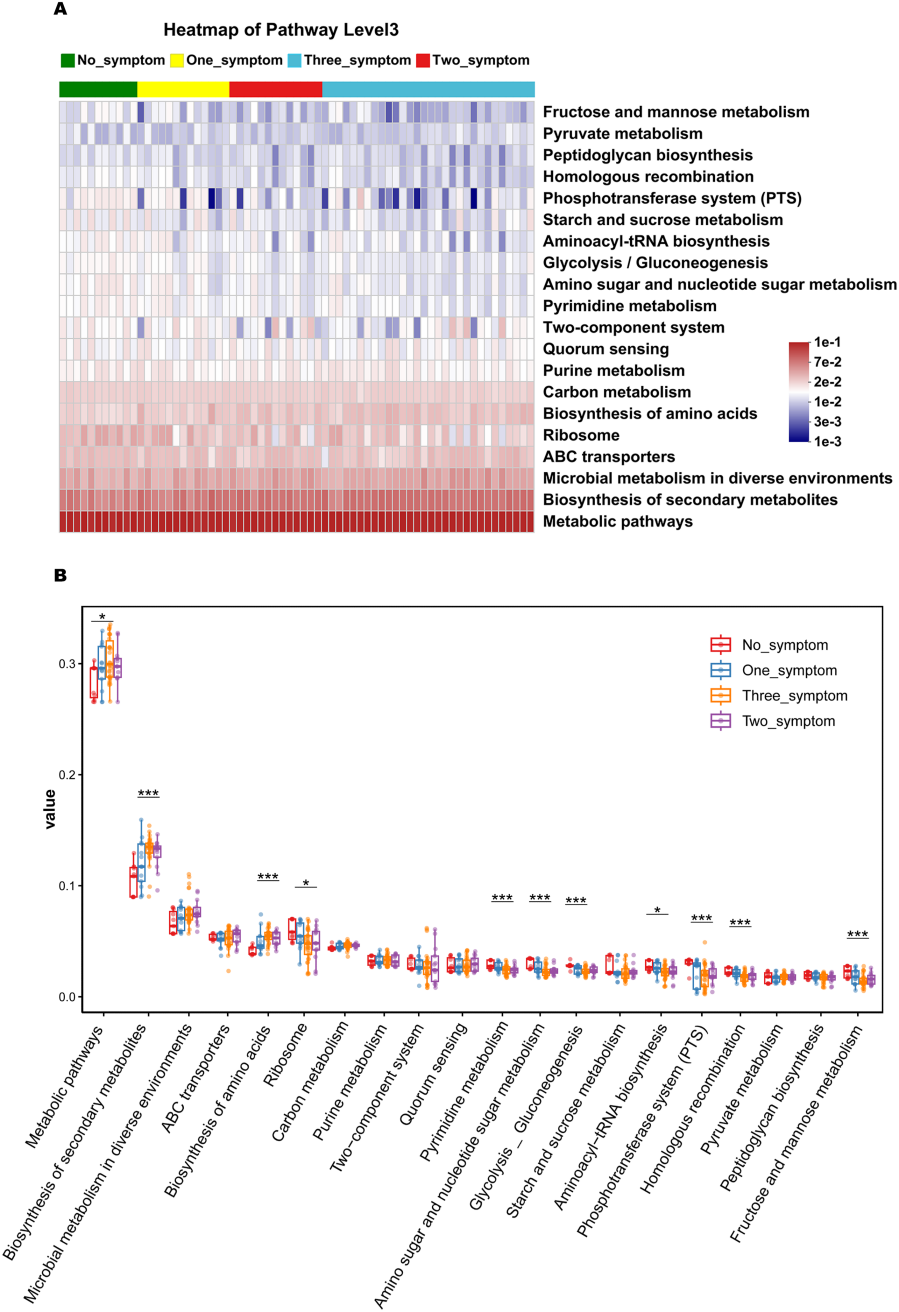
The predicted function capacity of the vaginal microbiome in GSM women with different types of symptoms. The KEGG pathways of the top 20 bacteria in relative functional abundance in the vaginal microbiome at level 3 were identified **(A)** and compared **(B)** in the no_symptom, one_symptom, two_symptom, and three_symptom groups. *P < 0.05, ***P < 0.001.

The potential functional capacity of the vaginal microbiome was predicted using PICRUSt2 based on the KEGG database and KEGG pathways at level 3. The top 20 bacteria with relative functional abundance in the vaginal microbiome were identified and compared in the no_symptom, one_symptom, two_symptom, and three_symptom groups (**Figures 9A, B**). There were significant differences in the relative functional abundance of the metabolic, ribosome, biosynthesis of secondary metabolites, biosynthesis of amino acids, pyrimidine metabolism, amino sugar and nucleotide sugar metabolism, glycolysis_gluconeogenesis, aminoacyl-tRNA biosynthesis, phosphotransferase system (PTS), homologous recombination, and fructose and mannose metabolism pathways in the four groups (**Figure 9B**, all P<0.05). Compared to the non-GSM women in the no_symptom group, the relative functional abundance of the secondary metabolite biosynthesis and amino acid biosynthesis pathways was significantly higher (all P<0.05), while the relative functional abundance of the pyrimidine metabolism, amino sugar and nucleotide sugar metabolism, glycolysis_gluconeogenesis, phosphotransferase system (PTS), homologous recombination, and fructose and mannose metabolism pathways was significantly lower, in the GSM women with two or three types of symptoms in the two_symptom and three_symptom groups. Furthermore, compared to the non-GSM women in the no_symptom group, the relative functional abundance of the ribosome and aminoacyl-tRNA biosynthesis pathways was significantly lower in the GSM women in the three_symptom group (all P<0.05). However, there was no significant difference between non-GSM women in the no_symptom group and GSM women with one type of symptom in the one_symptom group (P>0.05). These results demonstrate that there are some significant changes in the potential functional capacity of the vaginal microbiome between non-GSM and GSM patients with more than one type of symptom, which might be associated with the clinical manifestations of GSM and is worthy of being studied in the future.

### 3.8 Effects of LLCVU on women with GSM

Our studies showed that Lactobacillus had an increased abundance in non-GSM women compared with GSM women. Therefore, to verify the effects of Lactobacillus on the symptoms and signs of GSM, the clinical data of patients with GSM who were treated with a vaginal administration of LLCVU were analyzed. The patients with GSM enrolled were between 42 and 85 years old. The results showed that the number of GSM women with genital symptoms significantly decreased after LLCVU treatment (**Table 2**, P<0.01). Compared to the baseline scores, the FSFI scores significantly increased, while the VHISs and VSSs significantly decreased at 2 weeks after the first day of LLCVU treatment (**Table 2**, P<0.01). These results demonstrate that LLCVU can partly relieve genital symptoms and signs, and improve the sexual life of women with GSM in short-term observation. Excitingly, these results are in accordance with our previous data that a decrease in Lactobacillus in the vagina is especially associated with the types of genital and sexual symptoms in women with GSM.

**Table 2:** Changes in women with GSM after the treatment with Live Lactobacillus Capsule for Vaginal Use (LLCVU) (n=29).

	Baseline	2 weeks after treatment	P value
GSM symptoms(N, %)			
Genital symptoms	29(100.0)	21(72.4)	0.004^a^
Urological symptoms	22(75.9)	18(62.1)	0.395^a^
Sexual symptoms	27(93.1)	25(86.2)	0.670^a^
**FSFI (mean ± SD)**	14.893 ± 7.624	17.445 ± 8.503	0.001^b^
Desire	2.421 ± 1.836	2.545 ± 1.164	0.325 ^b^
Arousal	2.214 ± 1.449	2.228 ± 1.466	0.492 ^b^
Lubrication	2.928 ± 1.717	3.631 ± 2.021	0.021 ^b^
Orgasm	2.228 ± 1.328	2.462 ± 1.463	0.186 ^b^
Satisfaction	2.759 ± 1.016	3.462 ± 1.275	0.013 ^b^
Pain	2.345 ± 1.603	3.117 ± 1.815	0.019 ^b^
**VSS (mean ± SD)**	0.883 ± 0.416	0.469 ± 0.258	0.000 ^b^
Dryness	0.966 ± 0.865	0.207 ± 0.412	0.000 ^b^
Soreness	0.621 ± 0.820	0.276 ± 0.455	0.057 ^b^
Irritation	1.000 ± 0.802	0.621 ± 0.677	0.037 ^b^
Dyspareunia	1.310 ± 0.712	1.138 ± 0.693	0.143 ^b^
Vaginal discharge	0.517 ± 0.634	0.103 ± 0.310	0.002 ^b^
**VHIS(mean ± SD)**	1.172 ± 0.324	0.703 ± 0.373	0.000 ^b^
Vaginal secretions	1.103 ± 0.618	0.379 ± 0.562	0.000 ^b^
Vaginal epithelial integrity	0.690 ± 0.660	0.448 ± 0.686	0.052^b^
Vaginal epithelial surface thickness	0.966 ± 0.566	0.793 ± 0.559	0.153 ^b^
Vaginal color	1.621 ± 0.562	1.276 ± 0.650	0.016^b^
Vaginal PH	1.483 ± 0.785	0.621 ± 0.677	0.000 ^b^

a Fisher’s exact test.

b Mann-Whitney U test

VSS, vaginal symptoms score; VHIS, vaginal health index score; FSFI, female sexual function index; SD, standard deviation.

## 4 Discussion

In our study, we found that the richness and diversity of vaginal microbiota gradually increased from the reproductive to the postmenopausal stages. The gradual transition in the vaginal microbial community from healthy to GSM included a gradual shift to dysbiosis. The richness and diversity of vaginal microbiota were significantly higher in GSM women than in non-GSM women, and the occurrence of GSM symptoms may be associated with the changes in the vaginal microbial communities in perimenopausal and postmenopausal women. Different types of GSM symptoms may be related to different vaginal microbial communities in GSM women. Lactobacillus was found to be negatively associated with the onset, severity, and type of GSM while some bacteria, such as Escherichia-shigella, Anaerococcus, Finegoldia, Enterococcus, Peptoniphilus_harei, and Streptococcus, were found to be positively associated with these aspects of GSM. Lactobacillus treatment can at least partly relieve genital symptoms and signs, and improve the sexual life of GSM women in short-term observation, which was in accordance with our findings that a decrease in Lactobacillus in the vagina was especially associated with the types of genital and sexual symptoms in GSM women. Our results proved that the dysbiosis of vaginal microbiota probably plays an important role in the occurrence and development of GSM.

Studies showed that GSM manifestations may be present in 15% of premenopausal women (Gandhi et al., 2016), while these were present in at least 50-70% of postmenopausal women (Moral et al., 2018). The North American Menopause Society 2020 position statement stated that GSM symptoms affect 27%–84% of postmenopausal women (The NAMS 2020 GSM Position Statement Editorial Panel, 2020). In this small-size study, we found that 52.17% of the perimenopausal women and 100% of the postmenopausal women had GSM symptoms. It is well-understood that symptoms and signs of GSM result from estrogen deficiency in the female genitourinary tract in perimenopausal and postmenopausal women. All women experience estrogen deficiency after menopause, but not all women experience GSM. However, although symptoms and signs associated with GSM most often emerge in perimenopause and menopausal women, they also occur in reproductive women with multiple etiologies (2020; Shardell et al., 2021). In our study, we also found GSM symptoms can occur in reproductive women (**Table 1**). A cross-sectional analysis further found that there was no association between serum estrogen and GSM symptoms (Mitchell et al., 2017). Thus, estrogen deficiency does not fully elucidate the etiology of GSM, and it is necessary to explore additional factors that modify the risk for GSM.

After menopause, female sexual hormone level and vaginal environment change gradually followed by changes in the vaginal microbial community structure (Geng et al., 2020). In this study, we first investigated the vaginal microbiota of women of different ages using full-length bacterial 16S rRNA gene sequencing. We found that the richness and diversity of vaginal microbiota were significantly higher in the postmenopausal women than in the reproductive and perimenopausal women, which was in accordance with other studies (Shardell et al., 2021). France et al. found that postmenopausal women in the United States of America (USA) primarily had a high prevalence of community state type (CST) IV-C and a low prevalence of Lactobacillus spp.-dominant CSTs in the vagina (France et al., 2020), while reproductive women in eastern and southern African primarily had a high prevalence of CST III and a high prevalence of Lactobacillus spp.-dominant CSTs in the vagina, as analyzed by VALENCIA, a nearest centroid classification method for vaginal microbial communities based on composition (France et al., 2020). In our study, Lactobacillus dominance of the vaginal microbiota was also commonly detected in the reproductive women, while non-Lactobacillus dominance of the vaginal microbiota, which mainly belonged to CST IV-C according to the classification used by VALENCIA (France et al., 2020), was commonly detected in the postmenopausal women. We found that Lactobacillus proportion in the vagina gradually decreased from the perimenopausal stage to the late postmenopausal stage, while the proportion of other bacteria, such as Escherichia-shigella and Gardnerella, gradually increased. Other studies also showed that the composition of vaginal microbiota was dominated by Lactobacillus in reproductive women, while the composition was less likely dominated by Lactobacillus and was more likely to be predominantly composed of aerobic and anaerobic bacteria in perimenopausal and postmenopausal women (Hummelen et al., 2011; Brotman et al., 2014; Mitchell et al., 2021).

Studies have demonstrated that vaginal microbiota is of particular significance for gynecological and reproductive illnesses and potentially has some intimate connections with GSM (Geng et al., 2020). Some studies showed that women with vaginal atrophy had a lower proportion of Lactobacillus (Shen et al., 2016; Brotman et al., 2018), whereas other studies did not show this significant association (Mitchell et al., 2017, Mitchell et al., 2018). Brotman et al. found that a low relative abundance of Lactobacillus was associated with GSM symptoms, which included the vulvovaginal symptoms of burning on urination, painful sexual intercourse, vaginal discharge, and itching, soreness or burning sensations (Brotman et al., 2014). However, this study did not recruit a large number of women and women with GSM with different types of symptoms were not analyzed respectively (Brotman et al., 2014). Hummelen et al. tested the vaginal microbiomes of 32 women with GSM in the post-menopausal stage with genital symptoms of vaginal dryness and found there was an inverse correlation between Lactobacillus ratio and vaginal dryness (Hummelen et al., 2011). However, Mitchell et al. found that there was no association between non-Lactobacillus dominance and the genital and sexual symptoms of GSM in perimenopausal and postmenopausal women, including vaginal dryness, vulvovaginal itch/burn, vaginal discharge, vaginal pain with intercourse, and inability to have sex (Mitchell et al., 2017). Another study involving 30 GSM patients further demonstrated that improvement in the genital and sexual symptoms of GSM was more common in women who received oral estradiol and those with Lactobacillus-dominant vaginal microbiota as compared to placebo, but the differences were not statistically significant (Mitchell et al., 2018). Moreover, in that study, one woman whose GSM symptoms improved lost Lactobacillus dominance after placebo treatment, while two women whose symptoms did not improve gained Lactobacillus dominance over 8 weeks after placebo and estradiol treatment (Mitchell et al., 2018). The different results in these studies may be partially related to different study protocols, sample sizes, methods of symptom evaluation, and statistical analysis. Thus, the correlation between the symptoms and signs of GSM and vaginal flora is still full of ambiguity (Geng et al., 2020). In our study, we found that the onset, severity, and type of GSM symptoms may be associated with the changes in the vaginal microbial communities in perimenopausal and postmenopausal women. Lactobacillus was found to be negatively associated with the onset, severity, and type of GSM while some bacteria, such as Escherichia-shigella, Anaerococcus, Finegoldia, Enterococcus, Peptoniphilus_harei, and Streptococcus, were found to be positively associated with these aspects of GSM. These results correspond to previous studies that showed that Lactobacillus was positively correlated with a healthier vaginal condition as well as negatively correlated with more severe GSM (Gliniewicz et al., 2019; Geng et al., 2020). Bacterial vaginosis (BV) is characterized by loss of Lactobacilli and increases in Gardnerella vaginalis. Gardnerella and Streptococcus were reported to be strongly associated with BV (Onderdonk et al., 2016), which may cause vulvovaginal symptoms. Streptococcus can colonize the female genital tract and cause severe infectious diseases (Zhu et al., 2020). Streptococcus was found to have a prominent association with more serious GSM symptoms (Geng et al., 2020), and a distinct bacterial community state characterized by streptococcus was found to be associated with vulvovaginal atrophy (Brotman et al., 2018; Geng et al., 2020). Enterococcus was found to have a higher impact on the increase of the virulence of Gardnerella vaginalis, which contributed to a better understanding of the development of BV-associated biofilms (Castro et al., 2019). Xiao et al. found that Enterococcus might be associated with the recurrence of BV (Xiao et al., 2019). Hugenholtz et al. found that RUTI were predominantly caused by Escherichia_Shigella in perimenopausal women (Hugenholtz et al., 2022). Anglim et al. found that Finegoldia might be associated with RUTI in postmenopausal women (Anglim et al., 2022). Fok et al. found that Finegoldia was associated with the severity of urinary symptoms in women (Fok et al., 2018). Taken together, these findings indicate that GSM symptoms might be caused by an increase in these bacteria in perimenopausal and postmenopausal women and ecological interactions are important for the vaginal environment and reproductive health. Nevertheless, the potential interactions are far from definitive and these are areas for future study.

To address the multidimensionality of GSM, three types of GSM symptoms, i.e., genital, urological, and sexual symptoms, were investigated. Our study specifically addressed relationships between the types of GSM and vaginal microbiota. Our results demonstrate that Lactobacillus, Escherichia-shigella, Streptococcus, etc., may be associated with the types of genital and sexual symptoms in women with GSM in the perimenopausal and postmenopausal stages. Subsequently, diagnosis models for the identification of non-GSM women and GSM women were built using these vaginal bacteria. It was found that the diagnosis models built using these specific vaginal bacteria to differentiate between non-GSM women and GSM women with different types of GSM symptoms were comparatively accurate, especially in distinguishing between non-GSM women and GSM women with genital and sexual symptoms in the two_symptom and three_symptom groups, which might be useful for the prediction of different types and severity of GSM. However, there is also a limitation in our analysis in that our sample size is relatively small, which might partly limit the value of this research. We speculate that a study with a larger sample size is needed to further improve the accuracy of our prediction models, especially between non-GSM and GSM patients with one type of symptom. This limitation will be improved in our subsequent research.

The Lactobacillus species can produce lactic acids, lower pH, and inhibit the growth of bacteria and maintains an ideal vaginal environment and protects vaginal health (Amabebe and Anumba, 2018; Hassanein et al., 2023). In our study, we found that a shift in Lactobacillus-dominant vaginal microbiota to dysbiosis was possibly associated with GSM symptoms in perimenopausal and postmenopausal women and an underlying dysbiosis of the vaginal microbiome was believed to correspond with GSM symptomatology. It has been reported that 20% to 50% of postmenopausal women with a Lactobacillus-dominated vaginal microbiome had a lower prevalence of GSM symptoms (Park et al., 2023). Associations of Lactobacillus with GSM symptoms suggest a probable way to treat and prevent GSM. Thus, the effects of LLCVU, live Lactobacillus for vaginal use, on the treatment of GSM were analyzed. We found that LLCVU can partly relieve genital symptoms and signs, and improve the sexual life of GSM women, and the results are consistent with a pilot randomized controlled trial in which GSM symptoms were improved by using feminine hygiene products containing Lactobacillus in pre and postmenopausal women. However, according to the instructions for the use of LLCVU, it is mainly recommended to be used for the treatment of BV caused by bacterial dysbiosis. The bacteria we found to be correlated with GSM symptoms in our study, including Streptococcus and Enterococcus, are also strongly associated with the occurrence of BV (Onderdonk et al., 2016; Castro et al., 2019; Xiao et al., 2019), which may also explain the reason for the efficacy of LLCVU treatment for GSM from another perspective. However, our data for the efficacy of LLCVU for GSM was from short-term observation, and studies have shown that the application of a Lactobacillus ointment can improve the colonization of Lactobacillus in the internal and external genital areas of postmenopausal women up to 10 days after cessation of treatment (Nappi and Palacios, 2014; Yoshikata et al., 2022). A randomized controlled study designed with a multi-course of LLCVU and a long-term efficacy observation is needed for further study (Yoshikata et al., 2022).

This study took advantage of the third-generation PacBio sequencing technology to improve the taxonomic specificity and sensitivity of vaginal microbiota profiling as well as to reduce the risk of false positives (Martinez-Porchas et al., 2016; Stinson et al., 2019; He et al., 2020). Compared with the traditional sequencing of 16S rDNA, the third-generation PacBio sequencing technology used in our study detected the full-length bacterial 16S rRNA gene sequencing and provided accurate information on the vaginal bacteria at the species level, which may be optimal for vaginal microbiome sequencing due to its high-fidelity long reads and high performance.

There are some limitations in this study. First, our sample size was relatively small, and all the postmenopausal women were found to have GSM symptoms, which led to a lack of samples from postmenopausal women without GSM symptoms to analyze. Second, we were unable to analyze the vaginal microbiota of GSM women with one type of genital, sexual, or urological symptom, or with only one symptom separately due to the lack of sufficient samples, which might also have resulted in the AUC value of the predictive model being lower than 0.9 for the differentiation between non-GSM and GSM patients with one type of symptom in this study. Third, we did not focus on the sexual hormone levels in non-GSM and GSM women in the perimenopausal and postmenopausal stages. Fourth, our study of the effects of LLCVU on women with GSM was not a randomized controlled study and 16S rRNA data were not obtained from the individuals. Thus, a large-sample randomized controlled study is needed and these limitations will be improved in our future research.

In conclusion, this study explored the vaginal microecosystem and vaginal microbial structure in women of different ages as well as their differences in perimenopausal and postmenopausal women with or without GSM symptoms using full-length bacterial 16S rRNA gene sequencing and elucidated a particular association between vaginal microbiota and the clinical characteristics of GSM. The gradual transition in the vaginal microbial communities from healthy to GSM included a gradual shift to dysbiosis. The onset, severity, and type of GSM symptoms may be associated with changes in the vaginal microbial communities in perimenopausal and postmenopausal women. Lactobacillus may be negatively associated with the onset, severity, and type of GSM symptoms while some bacteria, such as Escherichia-shigella, Anaerococcus, Finegoldia, Enterococcus, Peptoniphilus_harei, and Streptococcus, may be positively associated with these aspects of GSM symptoms. Vaginal microbiota dysbiosis probably contributes to the occurrence and development of GSM symptoms, especially vaginal and sexual symptoms. Interventions targeted at changing the vaginal microbiota in perimenopausal and postmenopausal women could possibly improve their GSM symptoms. Lactobacillus used in the vagina may be as a possible non-hormonal treatment option for GSM women with genital and sexual symptoms. These findings are of great importance for the exploring of etiology and management of GSM. However, further details of the microbiological mechanisms in the progression of GSM need to be studied.

## Data Availability

The datasets presented in this study can be found in online repositories. The names of the repository/repositories and accession number(s) can be found in the article/**Supplementary Material**.
