# Supplementary material for: Associations of vaginal microbiota with the onset, severity, and type of symptoms of genitourinary syndrome of menopause in women
Source: Front Cell Infect Microbiol. 2024 Sep 24;14:1402389. doi: 10.3389/fcimb.2024.1402389 (PMC11458563; doi:10.3389/fcimb.2024.1402389)
Supplement: Supplementary file 1 [file Table1.docx]

**Appendix A**

**Atrophy symptom questionnaire**

A. Dryness: no lubrication or secretions noted on perineum or after wiping; for sexually active patient, loss of lubrication with coitus

□ None: no sensation of dryness

□ Mild: feels dryness episodically, dryness does not interfere with daily activities

□ Moderate: sensation of dryness most of the time, dryness does not interfere with activities of daily living

□ Severe: sensation of dryness all the time, dryness interferes with activities of daily living

B. Soreness: throbbing, pressure, fullness sensation in vagina that causes discomfort

□ None: no sensation of soreness

□ Mild: Feels soreness episodically, soreness does not interfere with activities of daily living

□ Moderate: Sensation of soreness most of the time, soreness does not interfere with activities of daily living

□ Severe: Sensation of soreness all the time, soreness interferes with activities of daily living

C. Irritation: sand paper–type feeling, uncomfortable with clothing or undergarments touching the perineum

□ None: no sensation of irritation

□ Mild: Feels irritation episodically, irritation does not interfere with activities of daily living

□ Moderate: Sensation of irritation most of the time; irritation does not interfere with activities of daily living

□ Severe: Sensation of irritation all the time, irritation interferes with activities of daily living

D. Dyspareunia: Pain with coitus at penetration or after coitus feels soreness in the vagina*

□ None: Comfortable coitus, pain or soreness is a seldom occurrence

□ Mild: episodically occurs, not all the time, occasionally must stop coitus

□ Moderate: Most of the time, minimal satisfaction from coitus, often must stop coitus

□ Severe: Occurs all the time, cannot enjoy coitus, often must stop coitus, occasionally postcoital bleeding. May be abstinent because of dyspareunia

E. Vaginal discharge: odorous and/or greenish-yellow secretions, spotting noted on undergarments or with wiping

□ None: none or minimal discharge

□ Mild: episodic moderate amount noted on undergarments, need to occasionally wear pad

□ Moderate: most of the time, need to wear pad but does not interfere with activities of daily living

□ Severe: constant, need to wear pad, irritation to perineum, occasionally bloody, interferes with activities of daily living

*Item D not complete for those not sexually active.

**Appendix B**

**Vaginal health assessment**

A. Vaginal secretions

□ No atrophy: normal, clear secretions noted on vaginal walls

□ Mild: superficial coating of secretions, difficulty with speculum insertion

□ Moderate: scant and covering entire vaginal vault, may need lubrication with speculum insertion to prevent

pain

□ Severe: none, inflamed, ulceration noted, need lubrication with speculum insertion to prevent pain

B. Vaginal epithelial integrity

□ No atrophy: normal

□ Mild: vaginal surface bleeds with scraping

□ Moderate: vaginal surface bleeds with light contact

□ Severe: vaginal surface has petechiae before contact and bleeds with light contact

C. Vaginal epithelial surface thickness

□ No atrophy: rugation and elasticity of vault

□ Mild: poor rugation with some elasticity noted of vaginal vault

□ Moderate: smooth, some elasticity of vaginal vault

□ Severe: smooth, no elasticity, constricts in upper one third of vagina or loss of vaginal tone (cystocele and rectocele)

D. Vaginal color

□ No atrophy: pink

□ Mild: lighter in color

□ Moderate: pale in color

□ Severe: transparent, either no color or inflamed

E. Vaginal pH

□ No atrophy: <5

□ Mild: 5-5.49

□ Moderate: 5.5-6.49

□ Severe: ≥6.5
