# Supplementary material for: Associations of vaginal microbiota with the onset, severity, and type of symptoms of genitourinary syndrome of menopause in women
Source: Front Cell Infect Microbiol. 2024 Sep 24;14:1402389. doi: 10.3389/fcimb.2024.1402389 (PMC11458563; doi:10.3389/fcimb.2024.1402389)
Supplement: Supplementary file 2 [file Table2.docx]

**Appendix A** — Female Sexual Function Index (FSFI)

| Question | Response Options |
| --- | --- |
| Q1: Over the past 4 weeks, how often did you feel sexual desire or interest? | 5 = Almost always or always  4 = Most times (more than half the time)  3 = Sometimes (about half the time)  2 = A few times (less than half the time)  1 = Almost never or never |
| Q2: Over the past 4 weeks, how would you rate your level (degree) of sexual desire or interest? | 5 = Very high  4 = High  3 = Moderate  2 = Low  1 = Very low or none at all |
| Q3. Over the past 4 weeks, how often did you feel sexually aroused (“turned on”) during sexual activity or intercourse? | 0 = No sexual activity  5 = Almost always or always  4 = Most times (more than half the time)  3 = Sometimes (about half the time)  2 = A few times (less than half the time)  1 = Almost never or never |
| Q4. Over the past 4 weeks, how would you rate your level of sexual arousal (“turn on”) during sexual activity or intercourse? | 0 = No sexual activity  5 = Very high  4 = High  3 = Moderate  2 = Low  1 = Very low or none at all |
| Q5. Over the past 4 weeks, how confident were you about becoming sexually aroused during sexual activity or intercourse? | 0 = No sexual activity  5 = Very high confidence  4 = High confidence  3 = Moderate confidence  2 = Low confidence  1 = Very low or no confidence |
| Q6. Over the past 4 weeks, how often have you been satisfied with your arousal (excitement) during sexual activity or intercourse? Response Options | 0 = No sexual activity  5 = Almost always or always  4 = Most times (more than half the time)  3 = Sometimes (about half the time)  2 = A few times (less than half the time)  1 = Almost never or never |
| Q7: Over the past 4 weeks, how often did you become lubricated (“wet”) during sexual activity or intercourse? | 0 = No sexual activity  5 = Almost always or always  4 = Most times (more than half the time)  3 = Sometimes (about half the time)  2 = A few times (less than half the time)  1 = Almost never or never |
| Q8. Over the past 4 weeks, how difficult was it to become lubricated (“wet”) during sexual activity or intercourse? | 0 = No sexual activity  1 = Extremely difficult or impossible  2 = Very difficult  3 = Difficult  4 = Slightly difficult  5 = Not difficult |
| Q9: Over the past 4 weeks, how often did you maintain your lubrication (“wetness”) until completion of sexual activity or intercourse? | 0 = No sexual activity  5 = Almost always or always  4 = Most times (more than half the time)  3 = Sometimes (about half the time)  2 = A few times (less than half the time)  1 = Almost never or never |
| Q10: Over the past 4 weeks, how difficult was it to maintain your lubrication (“wetness”) until completion of sexual activity or inter- course? | 0 = No sexual activity  1 = Extremely difficult or impossible  2 = Very difficult  3 = Difficult  4 = Slightly difficult  5 = Not difficult |
| Q11. Over the past 4 weeks, when you had sexual stimulation or intercourse, how often did you reach orgasm (climax)? | 0 = No sexual activity  5 = Almost always or always  4 = Most times (more than half the time)  3 = Sometimes (about half the time)  2 = A few times (less than half the time)  1 = Almost never or never |
| Q12: Over the past 4 weeks, when you had sexual stimulation or intercourse, how difficult was it for you to reach orgasm (climax)? | 0 = No sexual activity  1 = Extremely difficult or impossible  2 = Very difficult  3 = Difficult  4 = Slightly difficult  5 = Not difficult |
| Q13: Over the past 4 weeks, how satisfied were you with your ability to reach orgasm (climax) during sexual activity or intercourse? | 0 = No sexual activity  5 = Very satisfied  4 = Moderately satisfied  3 = About equally satisfied and dissatisfied  2 = Moderately dissatisfied  1 = Very dissatisfied |
| Q14: Over the past 4 weeks, how satisfied have you been with the amount of emotional closeness during sexual activity between you and your partner? | 0 = No sexual activity  5 = Very satisfied  4 = Moderately satisfied  3 = About equally satisfied and dissatisfied  2 = Moderately dissatisfied  1 = Very dissatisfied |
| Q15: Over the past 4 weeks, how satisfied have you been with your sexual relationship with your partner? | 5 = Very satisfied  4 = Moderately satisfied  3 = About equally satisfied and dissatisfied  2 = Moderately dissatisfied  1 = Very dissatisfied |
| Q16: Over the past 4 weeks, how satisfied have you been with your overall sexual life? | 5 = Very satisfied  4 = Moderately satisfied  3 = About equally satisfied and dissatisfied  2 = Moderately dissatisfied  1 = Very dissatisfied |
| Q17: Over the past 4 weeks, how often did you experience discomfort or pain during vaginal penetration? | 0 = Did not attempt intercourse  1= Almost always or always  2 = Most times (more than half the time)  3 = Sometimes (about half the time)  4 = A few times (less than half the time)  5 = Almost never or never |
| Q18: Over the past 4 weeks, how often did you experience discomfort or pain following vaginal penetration? | 0 = Did not attempt intercourse  1 = Almost always or always  2 = Most times (more than half the time)  3 = Sometimes (about half the time)  4 = A few times (less than half the time)  5 = Almost never or never |
| Q19. Over the past 4 weeks, how would you rate your level (degree) of discomfort or pain during or following vaginal penetration? | 0 = Did not attempt intercourse  1 = Very high  2 = High  3 = Moderate  4 = Low  5 = Very low or none at all |

**Appendix B**—Scoring System

The individual domain scores and full scale score of the FSFI are derived by the computational formula outlined in the table below. Individual domain scores are obtained by adding the scores of the individual items that comprise the domain and multiplying the sum by the domain factor (see below). The full scale score is obtained by adding the six domain scores. It should be noted that within the individual domains, a domain score of zero indicates that no sexual activity was reported during the past month.

| Domain | Questions | Score Range | Factor | Minimum score | Maximum score |
| --- | --- | --- | --- | --- | --- |
| Desire | 1,2 | 1-5 | 0.6 | 1.2 | 6.0 |
| Arousal | 3,4,5,6 | 0-5 | 0.3 | 0 | 6.0 |
| Lubrication | 7,8,9,10 | 0-5 | 0.3 | 0 | 6.0 |
| Orgasm | 11,12,13 | 0-5 | 0.4 | 0 | 6.0 |
| Satisfaction | 14,15,16 | 0(or 1)-5 | 0.4 | 0 | 6.0 |
| Pain | 17,18,19 | 0-5 | 0.4 | 0 | 6.0 |
| Full Scale Score Range | | | | 2.0 | 36.0 |
