# Supplementary material for: Associations of vaginal microbiota with the onset, severity, and type of symptoms of genitourinary syndrome of menopause in women
Source: Front Cell Infect Microbiol. 2024 Sep 24;14:1402389. doi: 10.3389/fcimb.2024.1402389 (PMC11458563; doi:10.3389/fcimb.2024.1402389)
Supplement: Supplementary file 3 [file Table3.docx]

**Appendix**

Many people leak urine some of the time. We are trying to find out how many people leak urine, and how much this bothers them. We would be grateful if you could answer the following questions, thinking about how you have been, on average, over the PAST FOUR WEEKS.

| **1.How often do you leak urine?** *(Tick one box)*  never□0  about once a week or less often□1  two or three times a week□2  about once a day□3  several times a day □4  all the time□5 |
| --- |

| **We would like to know how much you think leaks.**  **2.How much urine do you usually leak (whether you wear protection or not)?**  *(Tick one box)*  none□0  a small amount□2  a moderate amount□4  a large amount□6 |
| --- |

| **3.Overall, how much does leaking urine interfere with your everyday life?**  *Please ring a number between 0 (not at all) and 10 (a great deal)*  0 1 2 3 4 5 6 7 8 9 10  not at all a great deal |
| --- |

**ICIQ score: sum scores 1+2+3□□**

| **4.When does urine leak?** *(Please tick all that apply to you)*  never- urine does not leak□  leaks before you can get to the toilet□  leaks when you cough or sneeze□  leaks when you are asleep□  leaks when you are physically active/exercising□  leaks when you have finished urinating and are dressed□  leaks for no obvious reason□  leaks all the time□ |
| --- |
