# Supplementary material for: Associations of vaginal microbiota with the onset, severity, and type of symptoms of genitourinary syndrome of menopause in women
Source: Front Cell Infect Microbiol. 2024 Sep 24;14:1402389. doi: 10.3389/fcimb.2024.1402389 (PMC11458563; doi:10.3389/fcimb.2024.1402389)
Supplement: Supplementary file 4 [file Table4.docx]

**Appendix**

**The Menopause-Specific Quality of Life Questionnaire**

For each of the following items, indicate whether you have experienced the problem in the **PAST MONTH**. If you have, rate how much you have been ***bothered*** by the problem.

|  |  | Not at all  bothered | |  | | Extremely  bothered |
| --- | --- | --- | --- | --- | --- | --- |
|  |  |  |  | 0 1 2 3 4 5 6 | |  |
| 1. | HOT FLUSHES OR FLASHES | □ | □ | → | 0 1 2 3 4 5 6 | |
|  |  | No | Yes |  |  |  |
| 2. | NIGHT SWEATS | □ | □ | → | 0 1 2 3 4 5 6 | |
|  |  | No | Yes |  |  |  |
| 3. | SWEATING | □ | □ | → | 0 1 2 3 4 5 6 | |
|  |  | No | Yes |  |  |  |
| 4. | BEING DISSATISFIED WITH MY PERSONAL LIFE | □ | □ | → | 0 1 2 3 4 5 6 | |
|  |  | No | Yes |  |  |  |
| 5. | FEELING ANXIOUS OR NERVOUS | □ | □ | → | 0 1 2 3 4 5 6 | |
|  |  | No | Yes |  |  |  |
| 6. | EXPERIENGING POOR MEMORY | □ | □ | → | 0 1 2 3 4 5 6 | |
|  |  | No | Yes |  |  |  |
| 7. | ACCOMPLISHING LESS THAN I USED TO | □ | □ | → | 0 1 2 3 4 5 6 | |
|  |  | No | Yes |  |  |  |
| 8. | FEELING DEPRESSED DOWN OR BLUE | □ | □ | → | 0 1 2 3 4 5 6 | |
|  |  | No | Yes |  |  |  |
| 9. | BEING IMPATIENT WITH OTHER PEOPLE | □ | □ | → | 0 1 2 3 4 5 6 | |
|  |  | No | Yes |  |  |  |
| 10. | FEELING OF WANTING TO BE ALONE | □ | □ | → | 0 1 2 3 4 5 6 | |
|  |  | No | Yes |  |  |  |
| 11. | FLATULENCE (WIND) OR GAS PAINS | □ | □ | → | 0 1 2 3 4 5 6 | |
|  |  | No | Yes |  |  |  |
| 12. | ACHING IN MUSCLES AND JOINTS | □ | □ | → | 0 1 2 3 4 5 6 | |
|  |  | No | Yes |  |  |  |
| 13. | FEELING TIRED OR WORN OUT | □ | □ | → | 0 1 2 3 4 5 6 | |
|  |  | No | Yes |  |  |  |
| 14. | DIFFICULTY SLEEPING | □ | □ | → | 0 1 2 3 4 5 6 | |
|  |  | No | Yes |  |  |  |
| 15. | ACHES IN BACK OF NECK OR HEAD | □ | □ | → | 0 1 2 3 4 5 6 | |
|  |  | No | Yes |  |  |  |
| 16. | DECREASE IN PHYSICAL STRENGTH | □ | □ | → | 0 1 2 3 4 5 6 | |
|  |  | No | Yes |  |  |  |
| 17. | DECREASE IN PHYSICAL STRENGTH | □ | □ | → | 0 1 2 3 4 5 6 | |
|  |  | No | Yes |  |  |  |
| 18. | FEELING A LACK OF ENERGY | □ | □ | → | 0 1 2 3 4 5 6 | |
|  |  | No | Yes |  |  |  |
| 19. | DRYING SKIN | □ | □ | → | 0 1 2 3 4 5 6 | |
|  |  | No | Yes |  |  |  |
| 20. | WEIGHT GAIN | □ | □ | → | 0 1 2 3 4 5 6 | |
|  |  | No | Yes |  |  |  |
| 21. | INCREASED FACIAL HAIR | □ | □ | → | 0 1 2 3 4 5 6 | |
|  |  | No | Yes |  |  |  |
| 22. | CHANGES IN APPEARANCE, TEXTURE OR TONE OF YOUR SKIN | □ | □ | → | 0 1 2 3 4 5 6 | |
|  |  | No | Yes |  |  |  |
| 23. | FEELING BLOATED | □ | □ | → | 0 1 2 3 4 5 6 | |
|  |  | No | Yes |  |  |  |
| 24. | LOW BACKACHE | □ | □ | → | 0 1 2 3 4 5 6 | |
|  |  | No | Yes |  |  |  |
| 25. | FREQUENT ORINATION | □ | □ | → | 0 1 2 3 4 5 6 | |
|  |  | No | Yes |  |  |  |
| 26. | INVOLUNTARY URINATION WHEN LAUGHING OR COUGHING | □ | □ | → | 0 1 2 3 4 5 6 | |
|  |  | No | Yes |  |  |  |
| 27. | CHANGE IN YOUR SEXUAL DESIRE | □ | □ | → | 0 1 2 3 4 5 6 | |
|  |  | No | Yes |  |  |  |
| 28. | VAGINAL DRYNESS DURING INTERCOURSE | □ | □ | → | 0 1 2 3 4 5 6 | |
|  |  | No | Yes |  |  |  |
| 29. | AVOIDING INTIMACY | □ | □ | → | 0 1 2 3 4 5 6 | |
|  |  | No | Yes |  |  |  |
