# Supplementary material for: Associations of vaginal microbiota with the onset, severity, and type of symptoms of genitourinary syndrome of menopause in women
Source: Front Cell Infect Microbiol. 2024 Sep 24;14:1402389. doi: 10.3389/fcimb.2024.1402389 (PMC11458563; doi:10.3389/fcimb.2024.1402389)
Supplement: Supplementary file 5 [file Table5.docx]

**Supplemental table 5. Clinical characteristics of non-GSM and GSM women with different types of symptoms in perimenopausal and postmenopausal status (n=67)**

| **Characteristics** | **No-symptom (n=11)** | **One-symptom (n=13)** | **Two-symptom (n=13)** | **Three-symptom**  **(n=30)** | **P** |
| --- | --- | --- | --- | --- | --- |
| **Age（Median，range）** | 45.5（40~51） | 49.5（40~59） | 60.5（48~76） | 60.9(47~79) | 0.000 ^a^ |
| **BMI（Median，range）** | 22.1(18.6~30.8) | 21.8(18.7~24.2) | 21.4(18.5~25.0) | 22.6(17.7~27.5) | 0.519 ^a^ |
| **Gravidity(Median，range）** | 2.9（1~4） | 2.5（2~4） | 2.3(1~4) | 2.9(1~7) | 0.321 ^a^ |
| **Parity（Median，range）** | 1.5(1~2) | 1.2(1~2) | 1.2(1~2) | 1.3(1~3) | 0.443 ^a^ |
| **Smoker status (%)** | 0 | 0 | 0 | 0 | — |
| **Drinker status (%)** | 0 | 0 | 0 | 0 | — |
| **MENOQL (mean±SD)** | 6.316±1.862 | 6.864±2.831 | 10.744±2.435 | 11.478±2.668 | 0.000 ^a^ |
| Vasomotor | 1.788±0.860 | 1.897±1.363 | 1.897±1.384 | 2.000±1.511 | 0.983 ^a^ |
| Psychosocial | 1.402±0.514 | 1.164±0.451 | 1.670±0.679 | 1.790±0.752 | 0.014 ^a^ |
| Physical | 1.398±0.507 | 1.366±0.232 | 1.920±0.596 | 2.099±0.525 | 0.000 ^a^ |
| Sexual | 1.727±0.757 | 2.436±1.658 | 5.256±2.187 | 5.589±1.637 | 0.000 ^a^ |
| **FSFI (mean±SD)** | 29.600±1.275 | 22.508±8.419 | 11.946±8.603 | 9.760±6.854 | 0.000 ^a^ |
| Desire | 4.691±0.451 | 3.692±0.975 | 2.215±0.961 | 1.920±0.729 | 0.000 ^a^ |
| Arousal | 4.500±0.684 | 3.415±1.704 | 1.731±1.574 | 1.380±1.301 | 0.000 ^a^ |
| Lubrication | 5.536±0.338 | 3.923±1.871 | 1.938±1.803 | 1.620±1.635 | 0.000 ^a^ |
| Orgasm | 4.582±0.209 | 3.508±1.651 | 1.815±1.748 | 1.373±1.392 | 0.000 ^a^ |
| Satisfaction | 4.764±0.216 | 4.154±0.902 | 2.523±1.124 | 2.187±0.877 | 0.000 ^a^ |
| Pain | 5.527±0.432 | 3.815±1.888 | 1.723±1.644 | 1.280±1.359 | 0.000 ^a^ |
| **VSS（mean±SD）** | 0.018±0.060 | 0.077±0.130 | 0.615±0.420 | 0.820±0.529 | 0.000 ^a^ |
| Dryness | 0 | 0 | 0.615±0.506 | 1.167±0.791 | 0.000 ^a^ |
| Soreness | 0 | 0 | 0.231±0.439 | 0.300±0.466 | 0.040 ^a^ |
| Irritation | 0 | 0 | 0.692±0.630 | 0.933±0.785 | 0.000 ^a^ |
| Dyspareunia | 0 | 0.231±0.439 | 1.385±1.044 | 1.533±1.042 | 0.000 ^a^ |
| Vaginal discharge | 0.091±0.302 | 0.154±0.376 | 0.154±0.376 | 0.167±0.379 | 0.947 ^a^ |
| **VHIS（mean±SD）** | 0.127±0.224 | 0.631±0.364 | 1.385±0.538 | 1.400±0.558 | 0.000 ^a^ |
| Vaginal secretions | 0.273±0.467 | 1.154±0.555 | 1.846±0.376 | 1.833±0.648 | 0.000 ^a^ |
| Vaginal epithelial integrity | 0 | 0.154±0.376 | 1.000±0.816 | 1.067±0.691 | 0.000 ^a^ |
| Vaginal epithelial surface thickness | 0.091±0.312 | 0.769±0.599 | 1.462±0.660 | 1.533±0.860 | 0.000 ^a^ |
| Vaginal color | 0.182±0.405 | 0.692±0.480 | 1.615±0.650 | 1.433±0.679 | 0.000 ^a^ |
| Vaginal PH | 0.091±0.312 | 0.385±0.650 | 1.000±0.913 | 1.133±0.819 | 0.001 ^a^ |
| **ICIQ-SF(mean±SD)** | 0 | 0.923±2.290 | 0 | 4.000±4.742 | 0.001 ^a^ |
| ICIQ-SF-question 1 | 0 | 0.231±0.599 | 0 | 0.967±1.217 | 0.001 ^a^ |
| ICIQ-SF- question 2 | 0 | 0.308±0.751 | 0 | 0.933±1.015 | 0.001 ^a^ |
| ICIQ-SF- question 3 | 0 | 0.385±0.961 | 0 | 2.100±2.708 | 0.001 ^a^ |

SD, standard deviation.

^a^Kruskal-Wallis nonparametric test.
