# Supplementary material for: Associations of vaginal microbiota with the onset, severity, and type of symptoms of genitourinary syndrome of menopause in women
Source: Front Cell Infect Microbiol. 2024 Sep 24;14:1402389. doi: 10.3389/fcimb.2024.1402389 (PMC11458563; doi:10.3389/fcimb.2024.1402389)
Supplement: Supplementary file 6 [file Table6.docx]

**Supplemental table 6-1 Pairwise PERMANOVA analyses among the reproductive, peri_menopausal, early_ post_menopausal and late_post_menopausal groups.**

| **pairs** | **R2** | **p.value** | **p.adjusted** |
| --- | --- | --- | --- |
| Early_post_menopausal vs Late_post_menopausal | 0.01284287 | 0.546 | 0.5460 |
| Early_post_menopausal vs Peri_menopausal | 0.25072836 | 0.001 | 0.0020 |
| Early_post_menopausal vs Reproductive | 0.38289052 | 0.001 | 0.0020 |
| Late_post_menopausal vs Peri_menopausal | 0.30363780 | 0.002 | 0.0030 |
| Late_post_menopausal vs Reproductive | 0.44595503 | 0.001 | 0.0020 |
| Peri_menopausal vs Reproductive | 0.04083217 | 0.123 | 0.1476 |

**Supplemental table 6-2 Pairwise PERMANOVA analyses among the reproductive, peri_menopausal_asymptomatic, peri_menopausal_symptomatic and post_menopausal groups.**

| **pairs** | **R2** | **p.value** | **p.adjusted** |
| --- | --- | --- | --- |
| Peri_menopausal_asymptomatic vs Peri_menopausal_symptomatic | 0.02352592 | 0.510 | 0.5100 |
| Peri_menopausal_asymptomatic vs Post_menopausal | 0.32689585 | 0.001 | 0.0030 |
| Peri_menopausal_asymptomatic vs Reproductive | 0.03089526 | 0.334 | 0.4008 |
| Peri_menopausal_symptomatic vs Post_menopausal | 0.22357673 | 0.002 | 0.0040 |
| Peri_menopausal_symptomatic vs Reproductive | 0.05597770 | 0.116 | 0.1740 |
| Post_menopausal vs Reproductive | 0.42782303 | 0.001 | 0.0030 |

**Supplemental table 6-3 Pairwise PERMANOVA analyses among the no_symptom, one_symptom, two_symptom and three_symptom groups.**

| **pairs** | **R2** | **p.value** | **p.adjusted** |
| --- | --- | --- | --- |
| No_symptom vs One_symptom | 0.07067797 | 0.198 | 0.2376 |
| No_symptom vs Three_symptom | 0.46858031 | 0.001 | 0.0030 |
| No_symptom vs Two_symptom | 0.19315089 | 0.024 | 0.0360 |
| One_symptom vs Three_symptom | 0.25683517 | 0.001 | 0.0030 |
| One_symptom vs Two_symptom | 0.11581408 | 0.014 | 0.0280 |
| Three_symptom vs Two_symptom | 0.04286015 | 0.321 | 0.3210 |
